# Comparative expression profiling reveals a role of the root apoplast in local phosphate response

**DOI:** 10.1186/s12870-016-0790-8

**Published:** 2016-04-28

**Authors:** Wolfgang Hoehenwarter, Susann Mönchgesang, Steffen Neumann, Petra Majovsky, Steffen Abel, Jens Müller

**Affiliations:** Proteome Analytics Research Group, Leibniz Institute of Plant Biochemistry, D-06120 Halle (Saale), Germany; Department of Stress and Developmental Biology, Leibniz Institute of Plant Biochemistry, D-06120 Halle (Saale), Germany; Department of Molecular Signal Processing, Leibniz Institute of Plant Biochemistry, D-06120 Halle (Saale), Germany; Institute of Biochemistry and Biotechnology, Martin Luther University Halle-Wittenberg, D-06120 Halle (Saale), Germany; Department of Plant Sciences, University of California-Davis, Davis, CA 95616 USA

**Keywords:** *Arabidopsis thaliana*, Phosphate deficiency, Root growth, Proteomics, Transcriptomics, Iron transport, Cell wall, Pectin

## Abstract

**Background:**

Plant adaptation to limited phosphate availability comprises a wide range of responses to conserve and remobilize internal phosphate sources and to enhance phosphate acquisition. Vigorous restructuring of root system architecture provides a developmental strategy for topsoil exploration and phosphate scavenging. Changes in external phosphate availability are locally sensed at root tips and adjust root growth by modulating cell expansion and cell division. The functionally interacting *Arabidopsis* genes, *LOW PHOSPHATE RESPONSE 1* and *2* (*LPR1/LPR2*) and *PHOSPHATE DEFICIENCY RESPONSE 2* (*PDR2*), are key components of root phosphate sensing. We recently demonstrated that the *LOW PHOSPHATE RESPONSE 1 - PHOSPHATE DEFICIENCY RESPONSE 2 (LPR1-PDR2)* module mediates apoplastic deposition of ferric iron (Fe^3+^) in the growing root tip during phosphate limitation. Iron deposition coincides with sites of reactive oxygen species generation and triggers cell wall thickening and callose accumulation, which interfere with cell-to-cell communication and inhibit root growth.

**Results:**

We took advantage of the opposite phosphate-conditional root phenotype of the *phosphate deficiency response* 2 mutant (hypersensitive) and *low phosphate response 1* and *2* double mutant (insensitive) to investigate the phosphate dependent regulation of gene and protein expression in roots using genome-wide transcriptome and proteome analysis. We observed an overrepresentation of genes and proteins that are involved in the regulation of iron homeostasis, cell wall remodeling and reactive oxygen species formation, and we highlight a number of candidate genes with a potential function in root adaptation to limited phosphate availability. Our experiments reveal that *FERRIC REDUCTASE DEFECTIVE 3* mediated, apoplastic iron redistribution, but not intracellular iron uptake and iron storage, triggers phosphate-dependent root growth modulation. We further highlight expressional changes of several cell wall-modifying enzymes and provide evidence for adjustment of the pectin network at sites of iron accumulation in the root.

**Conclusion:**

Our study reveals new aspects of the elaborate interplay between phosphate starvation responses and changes in iron homeostasis. The results emphasize the importance of apoplastic iron redistribution to mediate phosphate-dependent root growth adjustment and suggest an important role for citrate in phosphate-dependent apoplastic iron transport. We further demonstrate that root growth modulation correlates with an altered expression of cell wall modifying enzymes and changes in the pectin network of the phosphate-deprived root tip, supporting the hypothesis that pectins are involved in iron binding and/or phosphate mobilization.

**Electronic supplementary material:**

The online version of this article (doi:10.1186/s12870-016-0790-8) contains supplementary material, which is available to authorized users.

## Background

Inorganic phosphate (Pi) is an essential macronutrient for plant growth and development. Despite its high abundance in the rhizosphere, bioavailability of Pi is typically limited because its majority is bound in organic compounds or complexed with metal ions such as Ca (alkaline soils), Fe or Al (acidic soils) [1]. Thus, plants evolved strategies to enhance Pi acquisition and to conserve or remobilize Pi from internal sources to adapt to Pi limiting conditions. Previous efforts elucidated some of these adaptive responses, including the identification of high-affinity Pi transport systems, the characterization of diverse metabolic bypass reactions, the reutilization of Pi from phospholipids, and many more [2]. Most of the Pi starvation response (*PSR*) genes involved in these systemic adjustments are regulated by the myb transcription factor PHR1 (PHOSPHATE STARVATION RESPONSE1) [3–6].

Dynamic redesign of the root system architecture (RSA) provides another strategy to maintain cellular Pi supply. In *Arabidopsis*, low external Pi availability is locally sensed by the growing root tip, which causes reduction of cell elongation and meristematic activity at the site of Pi depletion. The resultant inhibition of root growth is accompanied by accelerated formation of root hairs and development of lateral roots to increase the absorptive surface for topsoil exploration [7, 8]. The development of a densely branched and/or shallow root systems increases Pi starvation tolerance in several plant species, including agronomically important crops such as barley, lupin, soybean or common bean [9]. Several *Arabidopsis* mutants with altered Pi dependent root growth responses have been described [10–18]. However, for most of the underlying genes only little information is available how they affect Pi sensing and root growth modulation. *LPR1* (*LOW PHOSPHATE ROOT1*), its closely related paralog *LPR2*, and *PDR2* (*PHOSPHATE DEFICIENCY RESPONSE2*) have been identified as central players in local root Pi sensing [11, 13, 19]. *PDR2*, which codes for the single P5-type ATPase of unknown substrate-specificity (AtP5A), and *LPR1*, encoding a multicopper oxidase, are expressed in overlapping domains of the root apical meristem (RAM). *LPR1* and *PDR2* interact genetically and are required for meristem maintenance and cell elongation in Pi-deprived roots. Importantly, the *lpr1lpr2* mutation impedes local root growth inhibition under Pi limitation and suppresses the hypersensitive short-root phenotype of *pdr2* plants, indicating that they act in the same pathway [11, 13].

Previous work revealed that external Fe availability modifies local Pi sensing [11, 13, 20]. A number of studies observed that Pi-starved *Arabidopsis* and rice plants accumulate elevated levels of Fe in the root and the shoot [20–23], which has been suggested as a proactive strategy to mobilize Pi from insoluble Fe complexes [8]. Fe participates in the formation of reactive oxygen species (ROS) and it has been proposed that Fe toxicity causes local root growth inhibition [20]. We recently provided evidence for apoplastic LPR1 ferroxidase activity and uncovered a major role of the *LPR1-PDR2* module for root tip-specific deposition of Fe^3+^ in cell walls (CW) of the RAM and elongation zone (EZ) during Pi limitation [19]. We further showed that Fe accumulation in the RAM is massively enhanced in Pi-starved *pdr2* roots, but suppressed in the insensitive *lpr1lpr2* line. Fe deposition coincides with sites of ROS generation and triggers CW thickening and callose accumulation, which interferes with cell-to-cell communication, RAM maintenance, and cell elongation.

In recent years, a set of transcriptome profiling studies provided significant insights into the transcriptional changes upon Pi deficiency in *Arabidopsis* [6, 21, 24–28]. In addition, a complementary transcriptome and proteome study highlighted the convergence of mRNA and protein expression profiles on lipid remodeling and glucose metabolism upon Pi-deprivation [25]. In this study, we performed comparative transcriptome and proteome expression profiling on roots of Pi-replete and Pi-starved wild-type (Col-0), *pdr2*, and *lpr1lpr2* plants in combination with a set of physiological and cell biological experiments. Our analysis emphasizes the importance of root Fe uptake and redistribution under Pi limitation. We highlight the potential role of so far unknown players in the regulation of Pi-dependent Fe-redistribution and demonstrate that apoplastic but not intracellular Fe accumulation triggers Pi-dependent root growth modulation. Consistently, we observed regulation of several CW modifying enzymes, which correlates with an increased deposition of pectin at sites of Fe accumulation. The potential role of pectin in Pi-dependent root Fe storage and Pi mobilization is discussed.

## Results

### Differential gene expression correlates with genotype-specific Pi sensitivity

**Fig. 1:**
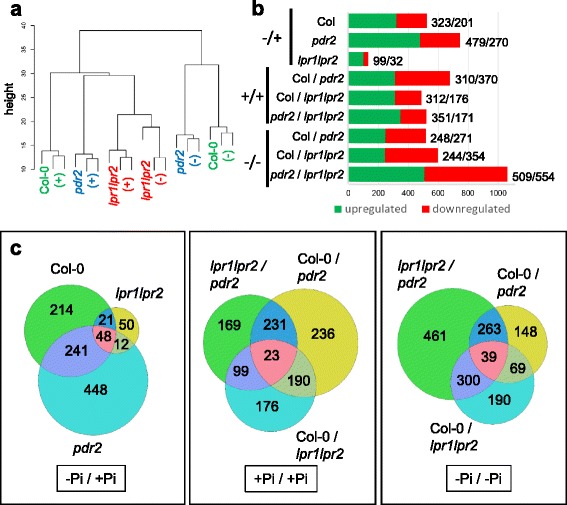
Statistical analysis of comparative gene expression analysis. **a** Hierarchical clustering of all ATH1 datasets, including wild-type (Col-0), *pdr2* and *lpr1lpr2* samples, the two growth regimes (+Pi and –Pi), and three biological replicates. Distances in the dendrogram illustrate the degree of relationship between samples. Note the short distance between biological replicate sets (*lowest branches*) compared to the relatively long distance between + Pi and –Pi conditions. **b** Number of up- and downregulated genes (*p* ≤ 0.05, Student’s *t*-test; 0.66 ≥ FC ≥ 1.5) in wild-type, *pdr2* and *lpr1lpr2* roots upon transfer from + Pi to –Pi (−/+), and in the three genotypes under + Pi (+/+) or –Pi (−/−) conditions. **c** Venn diagrams illustrating the number of differentially regulated genes in pairwise comparisons for all three genotypes under both growth regimes

For transcriptome analysis, wild-type, *pdr2* and *lpr1lpr2* seedlings were germinated on + Pi agar (4 days) and transferred to + Pi or –Pi medium for 20 h, a period during which Pi limitation alters global gene expression [28] as well as root meristem activity [19]. RNA was extracted from roots of three biological replicates and prepared for hybridization with ATH1 Affymetrix chips. Data were analyzed using ARRAYSTAR (Version 4.1.0) and further processed (Additional file 1: Table S1). Hierarchical clustering (Fig. 1a) confirmed high homogeneity within each replicate set because the biological replicates clustered together for each genotype and Pi condition (as indicated by the short branches at the bottom of the dendrogram). It also revealed a clear separation between + Pi and –Pi samples for the wild-type and the hypersensitive *pdr2* mutant (long branches between the + Pi and –Pi samples), but less pronounced differences for the insensitive *lpr1lpr2* line (shorter branches between the + Pi and –Pi samples). Pairwise comparisons using a fold-change cutoff value of ≥ 1.5 for increased and of ≤0.66 for decreased transcript levels (*p* ≤ 0.05; Student’s *t*-test) revealed 2292 differentially expressed genes across all genotypes and the two growth conditions. Low Pi exposure altered the expression of 749, 524, and 131 genes in *pdr2*, wild-type, and *lpr1lpr2* roots, respectively (Fig. 1b). Thus, the genotype-specific sensitivity of root growth inhibition in response to Pi depletion positively correlates with the number of differentially regulated genes.

### Identification of genotype-independent Pi-responsive genes

We generated Venn diagrams to illustrate the distribution of differentially expressed genes between the three genotypes (Fig. 1c). Wild-type shared a subset of 289 and 69 Pi-responsive genes with *pdr2* and *lpr1lpr2*, respectively, and all three lines had in common a core set of 48 genes (Fig. 1c). Hierarchical clustering of this core set revealed similar expression changes in all genotypes in response to –Pi with high positive correlation (Additional file 2: Figure S1 A, B). The core set comprises two partially overlapping groups that consist of at least 19 *PSR* and 23 metal-responsive genes (Table 1, Additional file 3: Table S2). Members of the first group (e.g., *SPX1*, *PAP17/ACP5*, *SRG3*, *CAX3*) are known targets of the Pi-regulated myb transcription factor PHR1 [5, 6, 29–31], suggesting that the systemic response to Pi deficiency is maintained in *pdr2* and *lpr1lpr2* mutants.

**Table 1:**
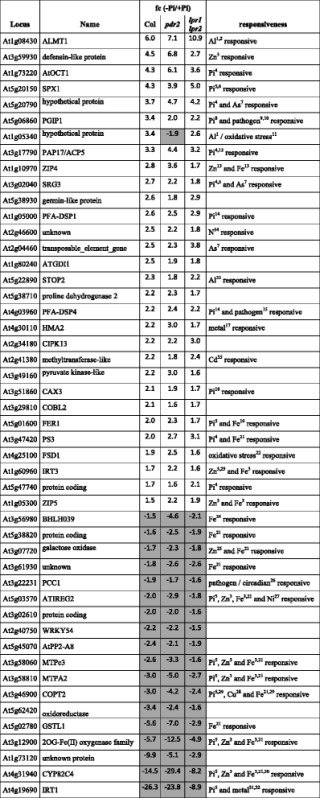
Pi-dependent transcriptional changes of commonly regulated genes

Shown is the fold change expression (FC) of all 48 Pi-responsive genes that are regulated in each of the tested genotypes (wild-type, *pdr2* and *lpr1lpr2*). Grey and white boxes denote genes that are significantly suppressed or induced, respectively (*p* ≤ 0.05, student’s *t*-test; 0.66 ≥ FC ≥ 1.5). All genes were interrogated for published responsiveness to Pi-starvation and/or metal-ions. References are indicated in superscript numbers and listed in Additional file 3: Table S2

In the second group, Fe-related genes are overrepresented (17 members) and comprise the majority of repressed genes (Table 1). The most strongly suppressed gene in all three genotypes (>10-fold repression) codes for IRT1, the major feedback-regulated Fe-uptake system in *Arabidopsis* [32, 33]. Many *IRT1* co-regulated genes (http://atted.jp) are induced under Fe deficiency [34–36]. Interestingly, 13 of the top 25 co-regulated genes are repressed in Pi-starved roots irrespective of the genotype (Table 2). Intriguingly, Pi-replete *pdr2* roots show higher expression of at least 12 Fe-related genes (Table 2), including a group of transcription factors (BHLH039, BHLH101, MYB10, MYB72) known to promote Fe-uptake under Fe deficiency [37–39]. The remaining Fe-related genes of this group are similarly induced in all three genotypes and encode the Fe storage protein FERRITIN1 (FER1) and various Fe-responsive metal transporters thought to be involved in transition metal detoxification and homeostasis (Table 1, Additional file 3: Table S2).

**Table 2:**
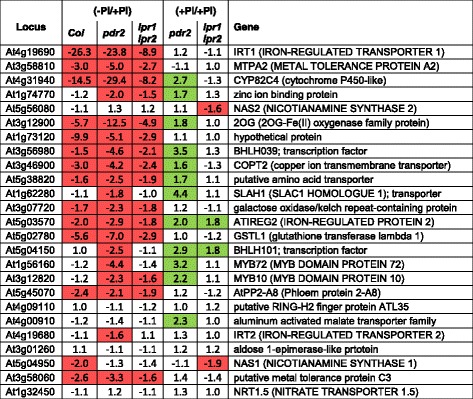
Pi-dependent regulation of the top 25 genes co-regulated with *IRT1* (ATTEDII)

Shown is the fold change expression in wild type, *pdr2* and *lpr1lpr2* after transfer to –Pi or the fold change expression of Pi-replete *pdr2* and *lpr1lpr2* plants compared to the wild-type. Red and green boxes denote genes that are significantly suppressed or induced (*p* ≤ 0.05, student’s *t*-test; 0.66 ≥ FC ≥ 1.5)

### Pi depletion alters expression of cell wall-related genes

We identified 241 Pi-responsive genes that are shared between the wild-type and the hypersensitive *pdr2* mutant, but not with the insensitive *lpr1lpr2* line (Fig. 1c). Surprisingly, only 10 genes of unknown functions in Pi starvation response were significantly deregulated in *pdr2* compared with the wild-type (>2-fold), whereas the remaining genes showed a high positive correlation (*r* = 0.88) between both genotypes (Additional file 2: Figure S1C, Additional file 4: Table S3). GO term analysis revealed high overrepresentation of gene products associated with the extracellular region (GO:0005576). An extended analysis for enriched GO terms within a group of 1680 genes (Additional file 5: Table S4), which are either regulated by –Pi in one or more genotypes or are differentially expressed in at least one of the lines in + Pi (*p* < 0.05; BH corrected), confirmed overrepresentation of genes (322) annotated to encode extracellular proteins (Additional file 2: Figure S1D, Additional file 6: Table S5). In this group, we identified a subset of 66 genes with putative functions in CW remodeling (Table 3). A similar number of genes were differentially expressed in *pdr2* (27) and wild-type (33) but only one-third (11) in *lpr1lpr2* roots. As noted for Fe-related genes, many CW-modifying genes (31) were deregulated in Pi-replete *pdr2* roots. Within the subset of 66 genes, 29 encoded proteins could be assigned a potential function in pectin modification, predominantly pectin methylesterification. In addition, we noted several expansins and xyloglucan endotransglycosylases (XTH) as well as a set of carbohydrate hydrolyzing enzymes. Intriguingly, all these proteins are predicted to regulate CW extensibility [40, 41].

**Table 3:**
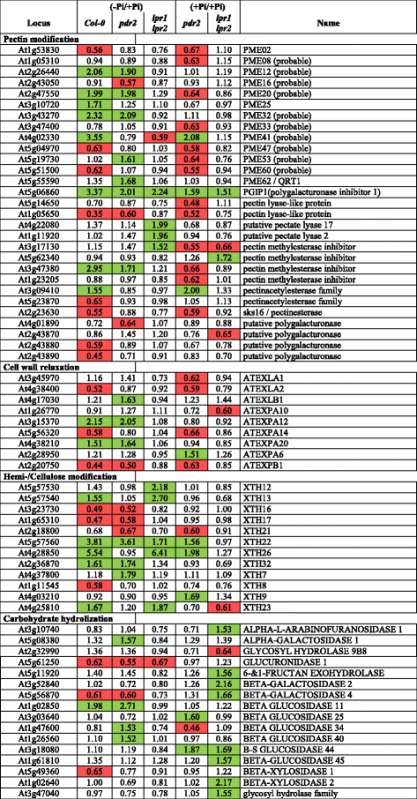
Pi-dependent regulation of cell wall modifying enzymes

Shown is the fold change expression of selected CW modifying enzymes in wild-type, *pdr2* and *lpr1lpr2* after transfer to –Pi or the fold change expression of Pi-replete *pdr2* and *lpr1lpr2* plants compared to the wild-type. Candidates were selected from a set of regulated genes annotated to be localized in the extracellular region (see also Additional File 6: Table S5). Red and green boxes denote significantly suppressed or induced (*p* ≤ 0.05, student’s *t*-test; 0.66 ≥ FC ≥ 1.5) genes. PME, pectin methyl esterase; EXP, expansin; EXL, expansin-like; XTH, xyloglucan endotransglucosylase/hydrolyse

GO term analysis also revealed overrepresentation of genes encoding tetrapyrrole- and heme-binding proteins (GO:0046906 and GO:0020037) with oxidoreductase activity (GO:0016491) (Additional file 2: Figure S1D). This group codes for 29 peroxidases and most of those (28) belong to the 73 member-family of class III peroxidases (CIII Prx) (Additional file 7: Table S6), which are extracellular enzymes with partly antagonistic functions in ROS formation and CW dynamics [42]. While Pi-responsive expression of 8 CIII Prx-encoding genes was similar between wild-type and *pdr2* roots, 7 genes were regulated independently in each line under low Pi, and only three CIII Prx genes responded significantly to Pi limitation in *lpr1lpr2* plants (Additional file 7: Table S6). Again, 19 CIII Prx genes were deregulated in *pdr2* under + Pi. Thus, peroxidases may be an important link between ROS formation and CW remodeling upon Pi starvation.

### Proteomics supports regulation of Pi-responsive genes in *pdr2* and *lpr1lpr2* mutants

Genotype-specific changes in the root proteome upon Pi deficiency were monitored in an unlabeled approach using a fast scanning high resolution accurate mass (HRAM) LC-MS system. Three biological and three technical replicates were measured for each genotype under + Pi and –Pi conditions (54 samples) yielding 3,328,368 MS/MS spectra (individual peptide measurements). 726,944 spectra could be annotated to a peptide sequence (peptide spectral match, PSM) with a global false discovery rate (FDR) threshold of 0.01 %. These PSMs were used to identify 5110 protein groups (unique proteins), each with at least one unique peptide and a global FDR threshold of 1 % (Additional file 8: Table S7). Protein abundance was inferred based on peptide abundance determined by peptide ion signal peak integration using the PROGENESIS software. Pairwise comparison of all genotypes under both growth regimes revealed 2439 differentially regulated proteins (*p* ≤ 0.05). Based on this list, we identified 1304 proteins that were either Pi-responsive in at least one genotype or which were already deregulated in one of the mutant lines grown on Pi-replete conditions (0.769 ≥ FC ≥ 1.3) (Additional file 9: Table S8).

**Fig. 2:**
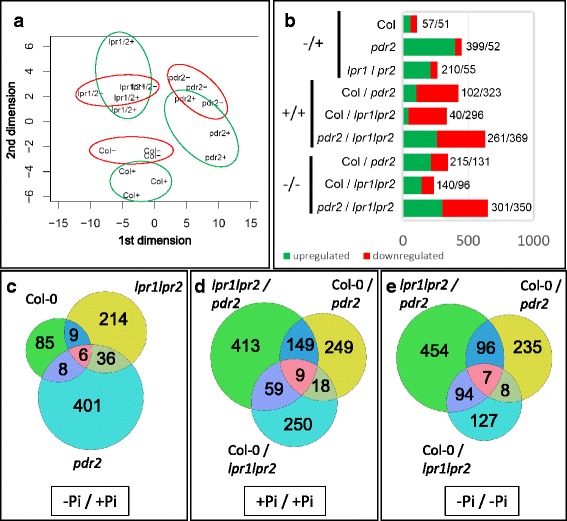
Statistics of comparative protein expression analysis. **a** Multidimensional scaling (MDS) analysis of all biological replicate samples. **b** Illustration of the number of up- and downregulated proteins (*p* ≤ 0.05, Student’s *t*-test; 0.769 ≥ FC ≥ 1.3) in wild-type, *pdr2* and *lpr1lpr2* roots upon transfer from + Pi to –Pi (−/+), and the number of differentially regulated proteins in pairwise comparisons of the three genotypes under + Pi (+/+) or –Pi (−/−) conditions. **c**, **d**, and **e** Venn diagrams illustrating the number of regulated proteins in pairwise comparisons for all three genotypes under both growth regimes (see also Additional file 8: Table S7, Additional file 9: Table S8, Additional file 10: Table S9)

Multidimensional scaling (MDS) analysis of ANOVA filtered (*p* < 0.05) samples revealed low variance between biological replicates but significant differences between genotypes and Pi conditions (Fig. 2a). The levels of 108 proteins were increased or decreased in the wild-type upon Pi depletion (Fig. 2b). As expected, the highest number of proteins (451) were regulated in hypersensitive *pdr2* mutant, probably reflecting changes in root morphology. We also identified a high number of Pi-responsive proteins (265) in the insensitive *lpr1lpr2* line. Of these, 214 proteins were unique to *lpr1lpr2* (Fig. 2c), indicating that the adjustment of protein expression might contribute to the decreased Pi responsiveness. Both mutant lines showed differential regulation of more than 300 proteins under Pi-replete conditions. This relatively high value is reminiscent of what we observed in the transcript dataset, supporting the assumption that PDR2 and LPR proteins may also regulate Pi independent processes.

Venn diagrams identified a group of 6 proteins that were similarly regulated in all lines upon Pi depletion (Fig. 2c, d, e). Notably, 4 of these proteins were positively correlated with our transcript data, showing induction on both mRNA and protein level (Table 4). Two members of this group were FER1 and the pectin modifying enzyme POLYGALACTURONASE INHIBITING PROTEIN1 (PGIP1) [43, 44], which further indicates that changes in Fe distribution and CW modification are associated with the response to low Pi.

**Table 4:**
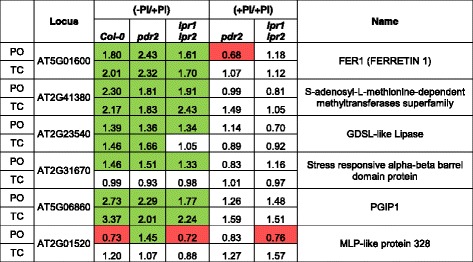
Pi-dependent Protein/mRNA regulation

Shown is the fold change expression of the 6 proteins (PO) that are Pi-responsive in all lines (wild-type, *pdr2* and *lpr1lpr2*) or the fold change expression of Pi-replete *pdr2* and *lpr1lpr2* plants compared to the wild-type. Protein expression is compared to transcript changes (TC). Red and green boxes denote genes that are significantly suppressed or induced (*p* ≤ 0.05, student’s *t*-test; 0.76 ≥ FC ≥ 1.3)

### Correlation of proteome and transcriptome analysis

Next, we performed GO term analysis to identify groups of proteins involved in genotype-specific Pi responsiveness. Most proteins have assigned metabolic functions in wild-type and *lpr1lpr2*, probably reflecting processes related to Pi recycling and mobilization. Strikingly, in + Pi condition and upon transfer to –Pi, the *pdr2* line showed a significant regulation of proteins assigned as *response to metal ion* (GO:0010038) and *oxidoreductase activity* (GO:0016491). A closer examination revealed repression of 15 peroxidases in *pdr2* in + Pi and induction of 9 peroxidases in –Pi condition. Within the group of repressed proteins we identified 14 CIII Prxs of which 3 enzymes were regulated at the transcript level. Only one and six Pi-responsive CIII Prx were detected in wild-type and *lpr1lpr2* root extracts, respectively (Additional file 10: Table S9).

**Fig. 3:**
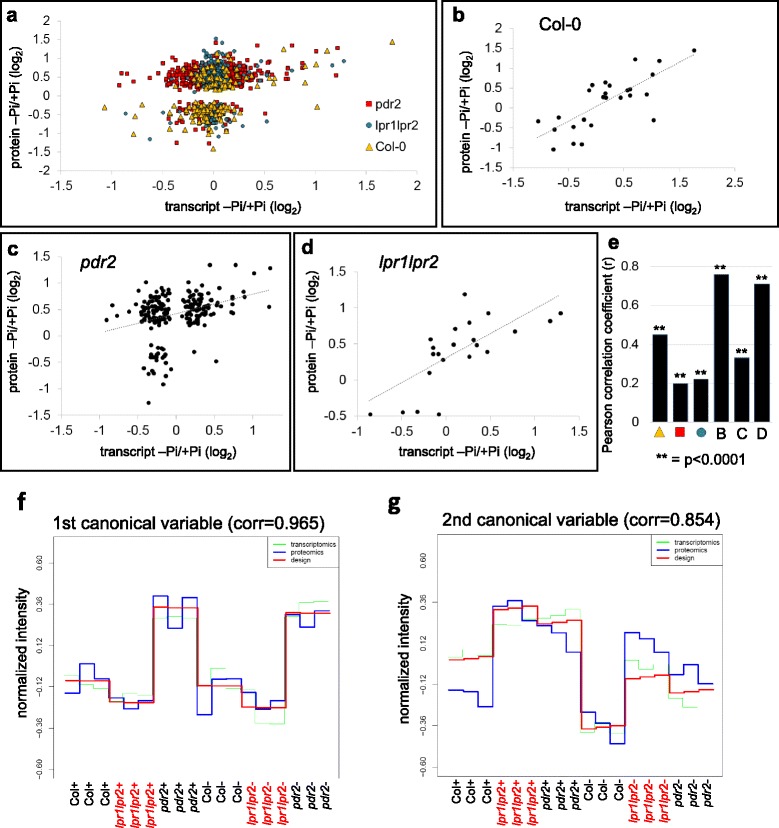
Comparative analysis of transcriptome and proteome data and spCCA analysis. **a**, **b**, **c**, and **d** Correlation between transcript and protein fold-changes upon Pi-deficiency. **a** For each genotype, all significantly regulated proteins (*p* ≤ 0.05) were plotted against its cognate transcript, if present on the ATH1 chip. **b**, **c**, and **d** Significantly (*p* ≤ 0.05) regulated protein/mRNA pairs were plotted against each other. Scatter plots show 26 protein/mRNA pairs regulated in wild-type upon Pi-deficiency, 211 pairs identified for *pdr2*, and 21 pairs identified in *lpr1lrp2*. **e** Correlation (r) values for each pairwise comparison. Asterisks indicate significance for each correlation analysis (*p* < 0.0001). **f**, **g** Canonical variables of the spCCA analysis representing a subset of transcripts/proteins which showed maximum correlation with the illustrated patterns that were generated by the spCCA algorithm. (see also Additional file 1: Figure S1, Additional file 11: Table S10. Additional file 13: Table S11)

To compare the proteome and transcriptome data sets, we plotted all significantly regulated proteins (*p* ≤ 0.05, Student’s *t*-test) against their cognate transcript. For those differentially expressed proteins, the percentage of detected transcripts was 91.6 % for wild-type (152/166), 94.3 % for *pdr2* (541/574) and 92.1 % for *lpr1lpr2* (351/381) roots. We observed only a low, but highly significant, positive correlation of transcript and protein abundance for all three genotypes (R ≥ 0.2, *p* ≤ 0.001) (Fig. 3a, b). We generated a list of significantly altered transcripts, which we compared to the list of significantly altered proteins (*p* < 0.05). We identified 26 cognate genes for wild-type, 22 for *lp1lpr2* and 211 for *pdr2*. The correlation coefficient markedly increased when we plotted these genes against their cognate proteins (Fig. 3b, c, d, e; Additional file 11: Table S10).

We identified the 4 genes, including *FER1* and *PGIP1*, that were co-regulated on mRNA and protein level across all genotypes in response to Pi depletion (Additional file 11: Table S10). In wild-type, we noticed induction of PPa4 (PYROPHOSPHORYLASE 4), a candidate for Pi mobilization, and PCK1 (PHOSPHOENOLPYRUVATE CARBOXYKINASE 1), which is involved in metabolic adjustment to Pi deprivation [45]. We further identified two hemicellulose modifying enzymes, XTH8 and XTH31 (XYLOGLUCAN ENDOTRANSGLUCOSYLASE/HYDROLASE), which were slightly decreased in low Pi. Interestingly, both enzymes were previously shown to be regulated by SIZ1 [46], a SUMO E3-ligase involved in Pi dependent root growth remodeling [47, 48].

GO term analysis of the 211 mRNA/protein pairs altered in *pdr2* revealed an overrepresentation of metabolic processes. The second most significant term (*response to metal ion*) is consistent with altered metal homeostasis in *pdr2* plants [19]. For example, we noticed induction of FER3 and proteins potentially involved in detoxification of metal ion-induced ROS formation, including several GLUTATHIONE-*S*-TRANSFERASEs (GSTs) (Additional file 11: Table S10). We also identified F6’H1 (feruloyl-CoA 6’-hydroxylase 1), which is involved in coumarin biosynthesis and Fe-mobilization in alkaline soils [49–51]. Our datasets revealed anti-correlation of F6’H1 expression in *pdr2*, showing elevated protein but decreased transcript levels in –Pi and an inverse relation in + Pi (Additional file 1: Table S1, Additional file 9: Table S8), which indicates stringent regulation of F6’H1 expression in *pdr2*. In addition, protein level of CCoAOMT1 (caffeoyl coenzyme A O-methyltransferase 1), which converts caffeoyl-CoA to feruloyl-CoA, the substrate of F6’H1 [52], was also elevated in *pdr2* (Additional file 9: Table S8). Thus, coumarin-mediated mobilization of Fe may be involved in Pi dependent Fe accumulation.

### Integrative spCCA analysis supports Pi-dependent metal redistribution

We integrated the two –omics approaches to uncover relationships that are supported by both individual datasets. We performed a supervised penalized canonical correlation analysis (spCCA), which searches for correlations between a set of transcripts and proteins [53]. The experimental design was integrated into the analysis to allow for biological interpretation of the derived canonical variables. The experimental factors (i.e., genotype, Pi condition, replicate sample) were provided as a binary matrix of design vectors that uniquely characterize each sample (Additional file 12: Figure S2). The supervised correlation approach seeks a linear combination of a feature subset from each -omics dataset that correlates maximally with a subset of experimental design factors. To maximize stringency, only varying transcripts and proteins were considered for spCCA. For transcriptomics, we choose a list of 1143 ANOVA filtered genes (*p* ≤ 0.05, var ≥ 0.12) and for proteomics a list of 47 proteins (*p* ≤ 0.05, var ≥ 0.4). Our analysis revealed distinct canonical variables (CVs), each representing a specific pattern correlating with a subset of proteins and/or transcripts. The first two CVs revealed structured patterns (Fig. 3f, g), while a third CV was disordered and therefore not further examined (Additional file 12: Figure S2B). The first CV mainly represented genes/transcripts (g/t) that were differentially expressed in *pdr2* compared to wild-type and *lpr1lpr2* independently of Pi status (Fig. 3f). We examined the top 100 g/t in this variable and found several Fe-related candidates (Additional file 13: Table S11), such as Fe chelate reductase 3 (FRO3) [54] and MYB10, which is required for growth in Fe deficiency [37]. MYB10 and MYB72 mediate Fe-dependent induction of NICOTIANAMINE SYNTHASE 4 (NAS4) [37], which is also present in this group. NAS proteins synthesize nicotianamine, a Fe-chelator essential for Fe-remobilization in the root [55]. We further identified a member of the ALUMINUM ACTIVATED MALATE TRANSPORTER (ALMT) family. It is of note that *ALMT1* is most highly induced in all three genotypes during Pi depletion (Table 1).

The second CV mainly represented g/t that were similarly expressed in Pi-replete *pdr2* and *lpr1lpr2* roots but slightly differed from the wild-type. In contrast to the first CV, the majority of these g/t were Pi responsive in all genotypes. As expected, we found several known Pi acquisition g/t, including SPX1, CAX3, the phosphate transporter PT2, and the Pi starvation-inducible inorganic pyrophosphatase 1 (Additional file 13: Table S11). Many other g/t are implicated in metal homeostasis, e.g., the Fe/Zn transporters IRT1 and IRT3, the Ni transporter IREG2, the Zn/Cd transporter HMA2 or the NA transporter YSL2, further supporting our observation that metal homeostasis is strictly controlled in all genotypes upon Pi starvation.

### Root growth inhibition in low Pi is independent of general Fe uptake and cellular storage

We previously reported that LPR1-dependent Fe accumulation and distribution in root tips controls RAM activity in response to low Pi [19]. Our comparative transcriptomics and proteomics analysis of entire roots revealed Pi-responsive expression of Fe-related genes, notably *FER1* and *IRT1* (Table 1, Table 4), which correlated with Fe overload in Pi-starved roots of the three genotypes under study [19] (Additional file 14: Figure S3). To further investigate the role of Fe during the local response of roots to Pi availability, we analyzed the impact of *FER1* and *IRT1* loss-of-function mutants on Fe-distribution and root growth inhibition upon Pi deprivation.

**Fig. 4:**
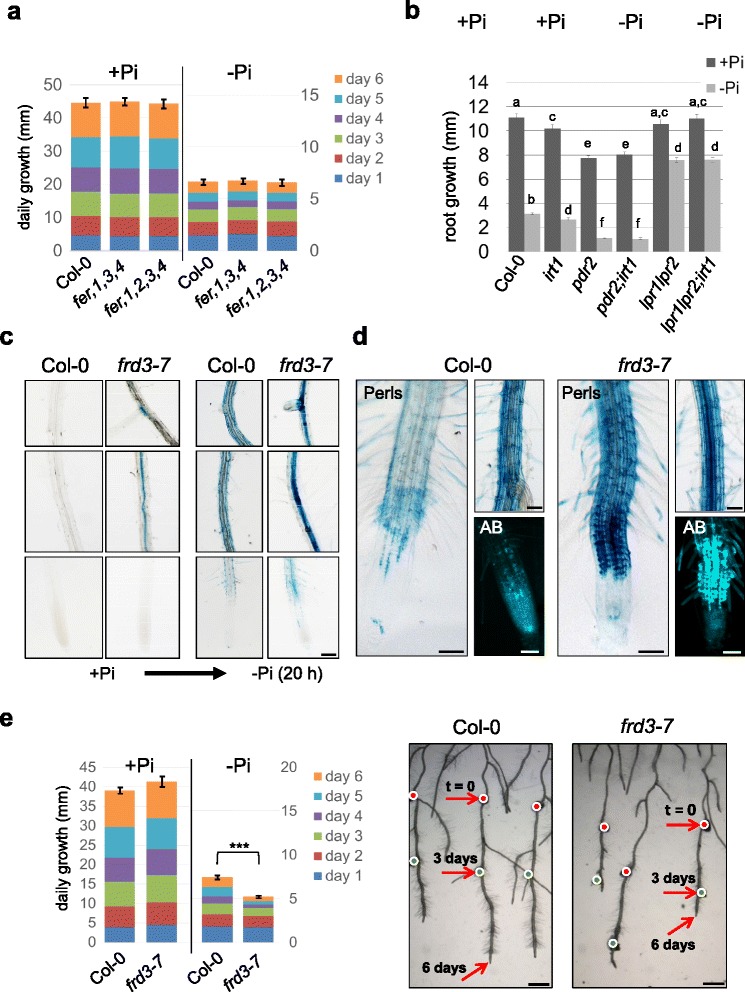
Root growth in *fer* and *irt1* mutant plants and phenotypes of *frd3* roots. **a** 4-days-old seedlings were transferred from + Pi to + Pi or –Pi medium for up to 6 days. Daily increase in root growth was measured and illustrated in segmented boxes within the bar graph (±SE, *n ≥* 15). Standard error was calculated from the average total root growth. **b** Total increase in root length after transfer from + Pi to either + Pi or –Pi medium *t*-test; *p* < 0.05 (±SE, *n* ≥ 20). **c**, **d**, and **e** Fe staining and root growth assays of wild-type and *frd3-7* seedlings. 4-days-old plants were transferred from + Pi to + Pi or –Pi medium for up to 6 days. **c** Perls staining in different root segments 20 h after transfer to + Pi or –Pi medium. Upper and middle panels show mature root segments. The lower panels show the RAM and early differentiation zone. Scale bar 200 μm. **d** Fe (Perls) and aniline blue (AB) callose staining of root tips and differentiated root segments 6 days after transfer to –Pi medium. Scale bar 100 μm. **e** Root growth of wild-type and *frd3-7* seedlings within 6 days after transfer to Pi-depleted medium. The bar graph shows the daily increase in root growth, illustrated in segmented boxes. Standard error was calculated from the average total root growth. *** *t*-test; p = 1.85^−8^ (±SE, *n ≥* 20). Overview images show the root growth after 3 days and 6 days on –Pi medium . Arrows indicate the position of the root tip, directly after transfer to –Pi (t = 0), as well as 3 days and 6 days after transfer. Scale bar 1000 μm. (See also Additional file 14: Figure S3)

Ferritins are located in plastids and can be visualized by Perls/DAB Fe staining as dot-like structures in root cells of wild-type plants, which are not detectable in *fer1-3-4* roots lacking *FER* expression [56]. Using semi-thin sections from Perls/DAB-stained wild-type roots, we observed similar dot-like structures in Pi-replete root tips, which strongly increased in number and staining intensity upon transfer to –Pi medium. These punctuate structures are associated with the symplast and are clearly distinctive from apoplastic Fe staining (Additional file 15: Figure S4A). We next performed root growth assays using the *fer1-3-4* triple and *fer1-2-3-4* quadruple mutant. Primary root growth rates of the *fer* mutants were indistinguishable from the wild-type on both + Pi or –Pi medium (Fig. 4a). Thus, ferritins do not affect the local root growth response to –Pi.

Similarly, we performed Perls/DAB Fe-staining to examine Fe distribution in wild type and *irt1* roots. Compared with Pi-replete wild-type seedlings, the *irt1* mutant showed more intense Fe staining on the root surface of the mature root zone (Additional file 15: Figure S4B), which is in accordance with impaired Fe uptake from the rhizosphere. However, both lines displayed similar Fe staining in the RAM and EZ, which is consistent with predominant *IRT1* expression in the differentiation zone [32] and confirms our previous study [19]. Under Pi depletion, Fe staining increased strongly and comparably in all segments of wild-type and *irt1* roots, indicating that Fe accumulation and distribution in root tips is independent of IRT1. We generated homozygous *pdr2irt1* double and *lpr1lpr2irt1* triple mutants and monitored primary root growth on + Pi and –Pi agar. As expected, the *irt1* mutation did not affect the Pi-dependent root growth response of *pdr2* and *lpr1lpr2* plants (Fig. 4b), indicating IRT1-independent Fe accumulation in the root tip in response to low Pi.

### Apoplastic Fe redistribution modifies Pi-dependent root growth adaptation

Long distance apoplastic Fe transport and distribution in symplastically disconnected tissues are mediated by the citrate exporter FERRIC REDICTASE DEFECTIVE 3 (FRD3) [57, 58]. Intriguingly, a previous study reported that *frd3* plants display a hypersensitive short-root phenotype when grown on –Pi medium [20]. To examine a potential role of FRD3 for mediating Pi-dependent Fe distribution via Fe-citrate complexes, we performed Perls Fe-staining (without DAB intensification to avoid oversaturation) on wild-type and *frd3* roots. As previously reported [58–60], Pi-replete *frd3* roots overaccumulated Fe in the vascular tissue (Fig. 4c). Within 20 h after transfer to –Pi, wild-type plants accumulated Fe in the outer cell layers, whereas *frd3* roots showed enhanced Fe staining in the vasculature, particularly in differentiated root segments. Importantly, only minor differences were noted in the root tip, where Fe accumulation was slightly increased in *frd3* (Fig. 4c); However, extended growth on –Pi (up to 6 days) progressively increased this difference, finally causing massive overaccumulation of Fe within the EZ and early differentiation zone of *frd3* roots (Fig. 4d).

We previously showed that Pi-dependent Fe accumulation correlates with callose formation at the sites of Fe deposition (<2 days) [19]. After transfer to –Pi (2 days), callose deposition at sites of Fe accumulation and resultant root growth inhibition were similar for wild-type and *frd3* plants (Additional file 16: Figure S5A). However, extended exposure (6 days) caused callose overproduction in *frd3* roots which correlated with an enhanced growth inhibition (Fig. 4d, e).

Based on our observations, we assumed that mobilization of apoplastic Fe-citrate complexes might be involved in the Pi dependent modulation of root growth. To test this, we transferred wild-type plants from + Pi conditions to + Pi or –Pi medium, supplemented with citrate, which was previously shown to restore Fe mobilization on *frd3* mutants [57] and monitored their growth behavior. Indeed, addition of 100–250 μM citrate promotes root growth within the first two days after transfer to –Pi. However, this effect was transient and external supply of citrate eventually suppressed root growth on low Pi (Additional file 16: Figure S5B, C).

### Pi deprivation modifies pectins at Fe accumulation sites

Our comparative expression profiling pointed to a role for pectin-modifying enzymes. Therefore, we studied Pi-dependent changes in the pectineous CW by using Ruthenium Red (RR), an inorganic dye that stains unesterified pectins [61, 62].

**Fig. 5:**
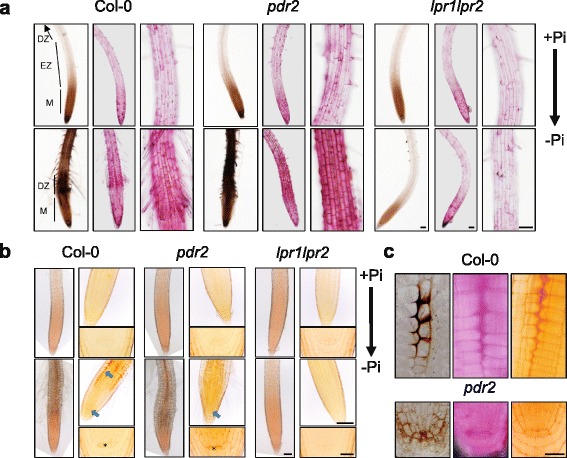
Fe and pectin staining of roots after seedling transfer from + Pi agar to + Pi or –Pi medium for up to 2 days. **a** Perls/DAB Fe staining (*left panels*) and Ruthenium Red (RR) staining of non-methylesterified pectins (*center and right panels*). Shown are overview images of Perls/DAB and RR stained roots as well as detail images of the elongation/early differentiation zone. Scale bar 100 μm. **b** Hydroxylamine ferric chloride (HFC) staining of methylesterified pectins. Shown are overview images of the root (scale bar 100 μm) as well as detail images of the root meristem (scale bar 100 μm) and the stem cell niche (scale bar 20 μm). Differences in coloration between overview and detail result from the use of different imaging devices. *Arrows* point to the SCN and cortical cell layer in the transition zone, asterisks label the quiescent center cells in the SCN. **c** Details of the Perls/DAB, RR and HFC staining in Pi-depleted cortical cells of Col plants and stem cells of *pdr2* plants. Scale bar 20 μm

Roots of wild-type, *pdr2* and *lpr1lpr2* showed a similar RR staining pattern on + Pi medium. One day after transfer to –Pi, we observed a strong increase in RR staining intensity in wild-type root tips, particularly within the differentiating EZ (Fig. 5a). Compared with wild-type, *pdr2* seedlings showed a more intense staining in this region while the RR staining in the *lpr1lpr2* mutant was unaltered. Interestingly, the site of enhanced pectin staining correlated well with the site of low Pi induced Fe deposition in wild-type and *pdr2* roots (Fig. 5a, c).

We also visualized the distribution of methyl-esterified pectin by using the hydroxylamine ferric chloride (HFC) reagent, which specifically reacts with methyl esters of pectin and results in a yellow to red coloration [62–64]. Only weak staining was evident in roots on + Pi (Fig. 5b) and transfer to –Pi did not significantly change the staining pattern in the differentiating EZ. However, higher magnification images revealed increased staining in the RAM of wild-type, with the highest intensity in the quiescent center (QC) and the cortical cell layer at the transition zone, which demarcates the border between the RAM and EZ. In contrast, *pdr2* seedlings showed enhanced staining in the RAM, particularly within the QC region, but no distinct labeling of the cortical cell layer. No differences in pectin staining were detected in *lpr1lpr2* roots after transfer to –Pi medium. High magnification images of RR- and HFC-stained roots revealed simultaneous accumulation of acidic and methyl-esterified pectin in the meristem of the two sensitive lines. In particular, after transfer to –Pi, strong HFC and RR staining was evident in the cortex cell layer of wild-type and in the QC region of *pdr2* roots (Fig. 5c), which co-localized with major sites of Fe deposition.

## Discussion

Plant adaptation to Pi limitation depends on coordinated transcriptional and translational regulation of gene expression [6, 21, 24–28]. While comparative transcriptome analysis proved to be a viable approach to distinguish between local and systemic regulation in Pi-starved plants [5, 6], only little information is available on the regulation of genes and proteins associated with the Pi-dependent adaption of root system architecture. Previous work revealed that *PDR2* and *LPR* genes act together in the local response to Pi availability by regulating cell type-specific deposition of Fe and callose in the root tip [11, 13, 19]. Here, we took advantage of the contrasting Pi-dependent root phenotype of *pdr2* and *lpr1lpr2* plants to investigate the associated changes in steady-state transcript and protein levels in a comparative approach. Genotype independent regulation of several *PSR* genes demonstrated the validity of our experiments and revealed that *pdr2* and *lpr1lpr2* mutants are likely not affected in the systemic response to Pi limitation (Table 1). Further analysis of our dataset revealed a number of candidate genes that are possibly involved in the Pi-dependent regulation of Fe storage and Fe redistribution as well as in the modulation of CW dynamics and/or ROS formation within the root.

### Pi depletion modulates root Fe distribution

Our study revealed genotype-independent repression of numerous Fe-responsive and *IRT1*-coregulated genes upon transfer to Pi limitation, which likely reflects feedback regulation as a consequence of elevated Fe accumulation in Pi-starved differentiated roots. On the other hand, de-repression of Fe-related genes in Pi-replete *pdr2* plants may sensitize Fe overaccumulation in limiting Pi [19] (Table 2, Additional file 12: Figure S2).

FER1 and related ferritins are plastid-localized Fe storage proteins protecting cells from Fe-mediated oxidative stress [65]. Using Perls/DAB Fe staining, Reyt et al. [56] recently reported dot-like structures in root cells of wild-type plants that likely display ferritin-bound Fe because they are absent in *fer1-3-4* roots [65]. A previous study showed that *FER1* is induced by PHR1 in low Pi independent of external Fe [66], indicating that FER1 may play a role in Pi-dependent Fe distribution.

Our comparative analysis revealed induction of FER1 expression on mRNA and protein level in all three lines (Table 4). Detection of Fe accumulation in dot-like structures supports the notion of intracellular Fe storage under Pi limitation, possibly as ferritin Fe (Figure S4). Importantly, Pi-dependent root growth was not affected by loss of ferritins (*fer1-[2]-3-4* mutants) or loss of *IRT1* in *pdr2* (*pdr2irt1)* and *lpr* (*lpr1lpr2irt1)* mutants (Fig. 4), indicating that Pi dependent root growth modulation is independent of intracellular Fe accumulation. Our data are consistent with a recent study reporting indistinguishable primary root growth of *fer1-3-4* and wild-type plants on high Fe [56].

Fe mobilization from the rhizosphere is facilitated by chelators such as carboxylates (e.g., citrate and malate) and coumarins, and apoplastic long distance Fe trafficking is mediated by Fe-citrate complexes [49–51, 67]. FRD3 exports citrate and the *frd3* mutant is defective in apoplastic Fe translocation, causing Fe hyperaccumulation in root stele tissues [58–60]. Importantly, *frd3* mutants show a hypersensitive short root phenotype in low Pi [20] and we demonstrated Fe overaccumulation in Pi-deprived *frd3* roots (Fig. 4), which indicates that citrate secretion is required for proper Fe-distribution under Pi limitation. Interestingly, citrate application transiently promoted *frd3* root growth in low Pi (Additional file 16: Figure S5), indicating that the Pi-dependent short root phenotype of *frd3* is likely a consequence of altered Fe redistribution in the growing root.

Transcript analysis and spCCA (Additional file 1: Table S1, Additional file 13: Table S11) revealed regulation of *ALMT* genes, including a strong Pi-dependent induction of *ALMT1* (Table 1), which was previously shown to exude malate into the rhizosphere as a strategy to cope with aluminum toxicity [68]. Earlier studies revealed PHR1-dependent accumulation of malate and citrate in Pi-depleted plants [24, 69]. Interestingly, exudation of both carboxylates into the rhizosphere was shown to facilitate mobilization of Pi and Fe in several plant species that do not form mycorrhiza [67].

We also noticed deregulation of coumarin biosynthesis-related genes, F6’H1 and CCoAOMT1, in *pdr2* roots (Additional file 1: Table S1, Additional file 9: Table S8). Several studies showed that coumarins (scopoletin and esculetin) are exuded into the rhizosphere to mobilize Fe in alkaline soils [49–51]. A recent report showed that esculetin accumulates in roots of Pi-starved wild-type plants but was suppressed in the *phr1* mutant, which lacks the induction of *PSR* genes upon Pi deficiency [69]. Moreover, using a non-targeted approach to identify metabolites from Pi-starved Arabidopsis root exudates, we recently confirmed Pi-dependent regulation of coumarin secretion [70]. Thus, our analysis implicates additional Fe-chelators in the regulation of Pi-dependent Fe accumulation and/or distribution in roots.

### Pi depletion modulates root pectins

Inhibition of root cell elongation, formation of root hairs and induction of lateral roots are the most robust local responses to Pi deficiency [7, 8], which all require extensive reorganization of the CW. Our analysis revealed Pi-dependent regulation of CW-modifying enzymes, particularly in the sensitive wild-type and *pdr2* plants and to a lesser extent in *lpr1lpr2* roots (Table 3). Consistent with a previous transcriptome study [28], we identified several putative pectin esterases and esterase inhibitors. Pectins are secreted into the apoplast in a highly methylesterified state. In the CW, pectin methylesterases (PME) may remove methyl groups, generating free carboxylate functions on the surface of pectin polymers. Crosslinking of these carboxylate-groups by Ca^2+^ reduces CW extensibility and regulates cell expansion [41]. Our experiments revealed low Pi-induced accumulation of non-methylated pectin, specifically within the EZ of wild-type and *pdr2* roots (Fig. 5), which might contribute to rapid inhibition of cell elongation in these lines. In addition, there is growing evidence that plants exchange Ca^2+^ ions for other divalent and trivalent metal ions to prevent metal uptake and ROS formation [71]. Gessa et al. [72] showed in vitro Fe^3+^ binding to carboxylate groups on polygalacturonic acids (PGA), and two studies in *Arabidopsis* and rice demonstrated the ability of PGA to mobilize Pi from FePO_4_ complexes and clay [73, 74]. Interestingly, a decrease in pectins in the *Arabidopsis qua1-2* mutant causes a hypersensitive short root phenotype upon Pi depletion [74]. Here, we show that accumulation of pectin in the root meristem coincides with the sites of Fe accumulation (Fig. 5a, c). Local pectin deposition might be a strategy to mobilize Pi from Fe-phosphate complexes. The data support our previous observations of CW thickening and callose deposition at sites of Fe accumulation in the root tip [19].

A recent study of the *Arabidopsis* flower transcriptome revealed deregulation of PGIP1 and other CW-modifying enzymes in the *ferritin1-3-4* triple mutant [75]. PGIP1 is a member of the leucine-rich repeat (LRR) protein superfamily and inhibits fungal and bacterial polygalacturonases, which cleave non-methylated pectin residues in infected tissues [43]. It further regulates germination by inhibiting the breakdown of seed coat pectins [44]. Intriguingly, our analysis revealed co-regulation of PGIP1 and FER1 on transcript and protein level in all lines upon Pi-depletion (Table 4), further indicating a potential link between the Pi-dependent regulation of Fe distribution and the modification of pectin in the CW.

### Peroxidases may modulate ROS formation and cell wall dynamics

We identified 41 CIII Prxs (56 % of the 73-member family) that were regulated on the mRNA and/or protein level, either in response to Pi depletion (23 members) or as a consequence of the *pdr2* and *lpr* mutations (Additional file 7: Table S6, Additional file 10: Table S9). Interestingly, the majority of CIII Prx mRNAs/proteins (30) were deregulated in *pdr2* in Pi replete conditions. CIII Prxs are involved in superoxide formation by transferring electrons from NADH to O_2_ as well as in the Fe catalyzed generation of hydroxyl radicals [76, 77]. ROS formation is likely responsible for the cleavage of CW polysaccharides to promote cell expansion. On the other hand, oxidation of monolignols by CIII Prxs is the predominant mechanism of monolignol polymerization (lignification) which rigidifies the CW and degrades H_2_O_2_ [78]. The potential role of CIII Prxs for modulating ROS levels and CW dynamics and their strong deregulation in *pdr2* mutants points to a function in local root growth adaptation. A comprehensive analysis of available transcriptome and proteome data revealed that most CIII Prxs are mainly expressed in the root [42]. Two of those, Prx33 and Prx34, bind to Ca^2+^ polygalacturonates and mediate root growth in *Arabidopsis* [79]. A more recent study demonstrated that *prx33* and *prx34* knock-down lines exhibited reduced ROS and callose formation upon treatment with microbe-associated molecular patterns (MAMPs), implicating a direct role of these gene products in ROS formation [80]. Using specific ROS indicators, we recently demonstrated the formation of apoplastic ROS at the site of –Pi induced Fe deposition [19]. The underlying mechanism remains elusive but CIII Prxs may constitute a missing link between Pi dependent ROS formation and CW remodeling.

### Comparative transcriptome and proteome analysis allows in-depth dissection of gene expression

Our comparative transcriptome and proteome analysis revealed a highly significant but relatively low positive correlation for the abundance of PSR proteins and their cognate transcripts in all three genotypes tested (Fig. 3a, e). The majority of mRNA/protein pairs in our dataset showed discordant changes, which has been previously observed and discussed in *Arabidopsis* and other organisms like mice and humans and which is likely explained by (post-) translational regulation and/or a temporal delay between the regulation of transcript and protein abundance. In addition, technical limitations in the efficiency of protein identification (e.g., low abundant proteins and transmembrane proteins) may restrict the detection of proteins relative to their cognate transcripts [25, 81–84].

Correlation values significantly increased when gene activity was subcategorized. For example, we observed a strong positive correlation between protein and mRNA abundance when we focused on proteins that were Pi-responsive in all genotypes (Table 4). Similarly, we found an enhanced positive correlation when we compared only significantly regulated genes with their cognate proteins (Fig. 3b, c, d, e). Moreover, our observations suggest that the integration of transcriptome and proteome datasets can be used as a valuable complementary approach. For example, we identified 28 and 18 CIII Prx, regulated on the transcript and/or protein level, respectively. Only 5 of those showed correlative expression changes in both datasets (Additional file 7; Table S6, Additional file 10: Table S9). However, the integration of both approaches revealed regulation of 41 CIII Prx, suggesting that the majority of CIII Prx are involved in the response to Pi deprivation.

We demonstrate that spCCA is a useful tool to integrate all experimental factors in our investigation, including the proteome and transcriptome data, Pi-status and genotype in order to elucidate unknown correlations in this multidimensional dataset. Interestingly, the first two CVs of our spCCA indicated a prominent role of genes and proteins that were differentially regulated in Pi-replete *pdr2* seedlings (Fig. 3f, g). Indeed, detailed analysis of our datasets revealed that the majority of Pi-responsive genes was not significantly deregulated in *pdr2*, compared to the wild type (Additional file 2: Figure S1C, Additional file 4: Table S3). On the other hand, several Fe-related genes, CIII Prx and pectin modifying enzymes were differentially regulated in Pi-replete *pdr2* plants (Table 2, Table 3, Additional file 7: Table S6), indicating that conditional hypersensitivity in *pdr2* might be a cause of constitutive de-repression or sensitization of these genes/proteins. P5-type ATPases are orphan, membrane localized ER proteins with unknown substrate specificity [85]. Mutant studies on yeast *SPF1* and *Arabidopsis MIA/PDR2* strongly suggest a function in ER quality control, protein folding and regulation of secretory processes [13, 86–88]. Hyperaccumulation of pectin and callose in the CW of Pi-depleted *pdr2* roots [19] (and this study) support a function of PDR2 in regulating ER-dependent secretion.

## Conclusions

We performed complementary transcriptomics and proteomics approaches to monitor changes in steady-state transcript and protein levels upon Pi deprivation of *Arabidopsis* wild-type, *pdr2* and *lpr1lpr2* roots. Our analysis reveals a set of genes and proteins that are involved in the regulation of Fe homeostasis, cell wall remodeling and ROS formation. We observed increased *FER1* and decreased *IRT1* expression in all genotypes, which are consistent with intracellular Fe accumulation and feed-back inhibited Fe uptake in Pi-depleted roots, respectively. Analysis of *fer1-3-4*, *fer1-2-3-4* and *irt1* mutants demonstrates that cellular Fe uptake and Fe storage in ferritin are not involved in Pi-dependent modulation of root growth. We provide evidence for the importance of apoplastic Fe redistribution to maintain root growth upon Pi-depletion and for a role of FRD3 in this process. Our data further reveal Pi-dependent regulation of cell wall-modifying enzyme expression and changes in the deposition of pectins in Pi-deprived roots. The high correlation between sites of Fe deposition and enhanced pectin accumulation suggests that pectins might be involved in Fe binding and/or Pi mobilization from Fe-P complexes.

## Methods

### Plant material and growth conditions

*Arabidopsis thaliana* accession Columbia (Col-0) and Col lines *pdr2-1*, *lpr1-1lpr2-1, irt1-1, frd3-7, fer1-3-4* and *fer1-2-3-4* were previously described [11, 13, 58, 89, 90]. The *pdr2-1* mutant was identified and characterized by our group [12, 13, 19]. The *irt1-1* (SALK_024525) and *frd3-7* (SALK_122235) lines were obtained from the European Arabidopsis Stock Center (NASC). The *lpr1-1lpr2-1* double mutant and the ferritin mutants (*fer1-(2)-3-4*) were kindly provided by T. Desnos [11] and J.F. Briat [90], respectively. Seeds were surface-sterilized and germinated on 0.8 % (w/v) Phyto-Agar (Duchefa) containing 50 μM Fe-EDTA and 2.5 mM KH_2_PO_4_, pH 5.6 (high or + Pi medium) or no Pi supplement (low or –Pi medium) as reported [13, 19].

### Root growth measurement

The position of the root tip was marked on the back of the agar plate directly after seedling transfer from + Pi to + Pi or –Pi medium. Images were taken on a stereomicroscope and total increment of primary root length was calculated at the according time point using ImageJ software. For daily growth rate measurements, the root tip position was marked every 24 h. The distance between two marker-points defines the daily root growth.

### Histochemical staining

Accumulation and distribution of Fe and callose in roots was monitored as previously described [19]. De-methyl esterified pectins were stained for 5–10 min in 0.05 % (w/v) Ruthenium Red solution (Applichem). Hydroxylamine-ferric chloride staining was adapted from Hornatowska and Reeve [63, 64]. Seedlings were initially incubated for 5–10 min in freshly prepared hydroxylamine solution (0.7 % NaOH, 0.7 % hydroxylamine hydrochloride in 60 % EtOH), followed by the addition of an equal (or higher) volume of a solution containing concentrated HCl/EtOH 95 % (1:2 ratio). The solution was removed and ferric chloride was added (10 % FeCl_3_ in 60 % EtOH containing 0.1 N HCl). Seedlings were cleared using chloral hydrate solution (7:7:1 chloral hydrate:ddH_2_O:glycerol). Samples were analyzed using a multizoom stereomicroscope (Nikon AZ100) for overview images and a Zeiss AxioImager bright field microscope for detail images.

### RNA preparation and microarray hybridization

Seedlings (4-days-old) were transferred from + Pi to either + Pi or –Pi medium and roots were harvested after 20 h. RNA was extracted using the RNeasy Plant Mini Kit from Qiagen followed by an on-column DNA digestion (40 min) using Qiagen RNase-free DNase Set. Quality control and hybridization to ATH1 *Arabidopsis* GeneChips was done by NASC’s Affymetrix Service (http://affymetrix.arabidopsis.info/).

### Statistical analysis of mRNA expression data

Data preprocessing, generation of Venn diagrams and heat maps was performed using Arraystar 4.1 software (DNASTAR). Arrays were normalized with robust multiarray analysis (RMA) and quantile background correction. Pairwise comparisons were performed using a fold-change cutoff value of ≥ 1.5 for increased and of ≤0.66 for decreased transcript levels (*p* ≤ 0.05; Student’s *t*-test, no multiple testing correction). Gene ontology analysis was done with the preassigned settings of the Arraystar software using a cutoff value *p* ≤ 0.05 and FDR (Benjamini Hochberg) correction. Hierarchical clustering was performed with 4870 ANOVA-filtered genes using the *hclust* package of the R software v.3.0.0. [91]. The ATTED-II database (http://atted.jp) was used to generate a list of *IRT1* co-regulated genes based on ATTED’s mutual ranking. All other calculations and graphics were prepared using Microsoft Excel 2010 software.

### Preparation of protein samples and LC-MS analysis

Plants were grown as for mRNA analysis. Proteins were extracted from root tissue and digested with trypsin. Peptides were injected into an EASY-nLC II nano liquid chromatography system, equipped with a Nanospray Flex ion source (Thermo Fisher Scientific) and electrosprayed into an Orbitrap Velos Pro mass spectrometer (Thermo Fisher Scientific). Details are described in Additional file 17.

### Protein identification and relative quantification

The raw data was imported into Proteome Discoverer v.1.4 (PD). Peak lists generated with a precursor signal to noise ratio of 1.5 with PD were used to search the TAIR10 database amended with common contaminants (35,394 sequences, 14,486,974 residues) with the Mascot algorithm v.2.5 on an in-house Mascot server. The enzyme specificity was set to trypsin and two missed cleavages were tolerated. Carbamidomethylation of cysteine was set as a fixed modification and oxidation of methionine as a variable modification. The precursor tolerance was set to 7 ppm and the product ion mass tolerance was set to 0.8 Da. A decoy database search was performed to determine the peptide false discovery rate (FDR). The search results were imported into the Scaffold Q+ software v.4.1.1 (Proteome Software, Inc.). Peptide and protein FDRs were calculated and the identity thresholds set to 0.01 and 1 % respectively to control the family wise error rate of peptide and protein identifications.

The raw data was imported into Progenesis LC-MS v.4.1 (Nonlinear Dynamics) for relative protein quantification between LC-MS analyses. The peptide ion signal peak landscapes of LC-MS analyses were aligned using the analysis as a reference that gave the highest minimum and maximum number of vectors in the aligned set of analyses when each analysis was used as a reference. Ratiometric normalization in log space to a selected reference analysis over all aligned peptide ion signals was performed. The summed intensities of peptide ion signal peak isotope envelopes over time were used as a measure of peptide abundance. A coefficient of variance (CV) of peptide abundance of less than 50 % for a peptide in all LC-MS analyses of a biological condition (three replicate analyses of each of three biological replicates for a total of 9) was required for a peptide to be quantified. Protein abundance was inferred by the sum of all unique peptides mapping to a given protein (non-conflicting peptides). Protein abundance fold changes and corresponding p-values between the biological conditions were calculated.

### Multidimensional scaling (MDS) analysis

Multidimensional scaling was conducted using the isoMDS function from the MASS package version 7.3-29 [92]. Technical replicates of the proteome analysis were averaged, reducing the original dataset to 18 biological replicate samples. Missing values were either imputed by half of the minimum intensity or excluded from further analysis. The resulting matrix of 3849x18 proteins was subjected to ANOVA (*p* < 0.05) revealing 412 consistent proteins. Intensities were log-transformed.

### Supervised penalized canonical correlation analysis (spCCA)

SpCCA analysis was done according to [53]. ANOVA filtered transcriptome and proteome data sets were reduced to signals with a variance of ≥0.12 and ≥0.4 resulting in 1143 transcripts and 47 proteins. The experimental design consisted of a binaric matrix of 18 samples x 8 experimental factors (three genotypes: Col, *pdr2*, *lpr1lpr2*; two growth media: +Pi, −Pi agar; and three replicates). SpCCA was conducted with 25 resampling runs (n.r = 25) and 25 random start vectors (max.counter.test = 25) to optimize sparsity parameters in a grid search between (0,0,0) and (0.6,0.5,1) with small step sizes (0.05,0.05,0.1) for transcriptomics, proteomics and design dataset.

### Ethics (and consent to participate)

Not applicable.

### Consent for publication

Not applicable.

### Availability of data and materials

Microarray data sets with the reference number NASCARRAYS-648 were deposited on the NASCArrays database (http://affymetrix.arabidopsis.info/). The proteomics data have been deposited to the ProteomeXchange Consortium [93] via the PRIDE partner repository with the dataset identifier PXD003449 and 10.6019/PXD003449 (http://www.ebi.ac.uk/pride/archive/).

## Additional files

Additional file 1: Table S1. ATH1 dataset. Shown is the relative average expression value of all probe sets (B-G) and the linear fold change of all pairwise comparions (L-AC). (XLSX 9833 kb) Additional file 2: Figure S1. Correlation and GO term analysis. (A) Heat map of a hierarchical cluster analysis of the group of 48 transcripts altered in all three genotypes upon Pi-depletion. Relative expression values are shown. (B) Scatter plots presenting pairwise correlation analysis (log_2_ fold changes) of the 48 commonly regulated genes upon Pi-depletion. FC, fold change. (see also Additional file 1: Table S1). (C) Correlation analysis of a subset of 241 Pi-responsive genes that were differentially regulated in wild-type and *pdr2* but not in *lpr1lpr2* roots (*p* ≤ 0.05, Student’s *t*-test; 0.66 ≥ FC ≥ 1.5). The upper image shows log_2_ fold changes of all genes upon Pi-starvation. The lower heat map illustrates the same gene set and expressional changes using a color code. (D) GO term analysis of a subset of 1680 genes that were either Pi-responsive in wild-type, *pdr2* and/or *lpr1lpr2* roots or that were differentially regulated in Pi-replete *pdr2* and/or *lpr1lpr2* roots (*p* ≤ 0.05, Student’s *t*-test; 0.66 ≥ FC ≥ 1.5). Each segment in a wheel represent one GO term. The top five GO terms are listed and significance values are shown. The complete list of genes and GO terms is shown in Additional file 4: Table S3. (see also Additional file 4: Table S3, Additional file 5: Table S4, Additional file 6: Table S5, Additional file 7: Table S6). (PDF 246 kb) Additional file 3: Table S2. Shown is a list of references that described Pi- or Fe-responsiveness of the genes listed in Table 1. (XLSX 14 kb) Additional file 4: Table S3. Pi-responsive genes exclusively regulated in wild-type and *pdr2* roots. Shown is the linear fold change of all 241 genes that showed Pi-dependent expressional changes (*p* ≤ 0.05, Student’s *t*-test; 0.66 ≥ FC ≥ 1.5) in wild-type and *pdr2* only, but not in *lpr1lpr2* roots. Highlighted are genes that were differentially expressed in *pdr2* (at least 2-fold higher or lower) compared to the wild-type. (XLSX 82 kb) Additional file 5: Table S4. Pi-responsive and deregulated genes in *pdr2* or *lpr1lpr2* roots and GO term analysis. Shown is a list of 1680 genes that were either regulated in one of the tested lines under Pi-depletion or differentially regulated in *pdr2* or *lpr1lpr2* in Pi-replete conditions, compared to the wild-type (*p* ≤ 0.05, Student’s *t*-test; 0.66 ≥ FC ≥ 1.5). Additional tabs show results from Gene Ontology analysis using the list of 1680 genes. BP, biological processes; MF, molecular function; CC, cellular compartment. (XLSX 1048 kb) Additional file 6: Table S5. Regulated genes of the GO term “extracellular region”. Listed are 322 genes whose encoded proteins are annotated to be located in the extracellular region (GO: 0005576) and which were either regulated in one of the tested lines under Pi-depletion or which were differentially regulated in *pdr2* or *lpr1lpr2* in Pi-replete conditions. This table is based on the list of 1680 genes (see Additional file 4: Table S3). (XLSX 98 kb) Additional file 7: Table S6. Regulation of extracellular peroxidases. Listed are 29 peroxidases that are annotated to be located in the extracellular region and found to be regulated either in one of the tested lines under Pi-depletion or which were differentially regulated in *pdr2* or *lpr1lpr2* in Pi-replete conditions. Green and red fields depict significantly induced or repressed genes, respectively (*p* ≤ 0.05, Student’s *t*-test; 0.66 ≥ FC ≥ 1.5). This table is based on the list of 322 regulated genes of the GO term “extracellular region” (see Additional file 6: Table S5). (XLSX 19 kb) Additional file 8: Table S7. Proteome data. Scaffold v4.4.1 was used to aggregate and visualize protein identifications from the Mascot search engine (v2.5.) run via Proteome Discoverer (V1.4) with X!Tandem searches integrated into Scaffold. LFDR scoring and protein cluster analysis for protein grouping were used to identify proteins. Total spectra (#PSMs) per protein normalized to the total spectra of all proteins recorded for each biological condition are shown. (XLSX 643 kb) Additional file 9: Table S8. Differentially regulated proteins. Listed are 1304 proteins that were either Pi-responsive in at least one genotype (Col-0, *pdr2* and/or *lpr1lpr2*) or which were already deregulated in one of the mutant lines grown on Pi-replete conditions (*p* ≤ 0.05, 0.769 ≥ FC ≥ 1.3). Green and red boxes represent proteins that were significantly induced or repressed, respectively. Blue boxes represent proteins that were significantly regulated (*p* ≤ 0.05) but did not reach the preassigned cut-off fold change value. (XLSX 230 kb) Additional file 10: Table S9. Regulation of peroxidases. (A) Listed are 23 peroxidases that were either Pi-responsive in at least one genotype (wild-type, *pdr2* and/or *lpr1lpr2*) or were already deregulated in one of the mutant lines grown on Pi-replete conditions (*p* ≤ 0.05, 0.769 ≥ FC ≥ 1.3). (B) Listed are 5 peroxidases that were regulated on transcript and protein level in at least one pairwise comparison. TC, transcript; PO, protein. Green and red boxes represent proteins which were significantly induced or repressed, respectively. (XLSX 60 kb) Additional file 11: Table S10. Regulation of mRNA/protein pairs. Listed are mRNA/protein pairs that showed correlative expression upon Pi-deficiency. Shown is a list of 26 pairs for wild-type, 211 pairs for *pdr2* and 22 pairs for *lpr1lpr2*. Green and red boxes represent proteins that were significantly induced or repressed, respectively (*p* ≤ 0.05, 0.769 ≥ FC ≥ 1.3). Blue boxes represent proteins which were significantly regulated (*p* ≤ 0.05) but did not reach the preassigned cut-off fold change value. (XLSX 73 kb) Additional file 12: Figure S2. Fe staining and root growth assay. Perls/DAB Fe staining on 4-days-old seedlings that were transferred from + Pi to + Pi or –Pi medium for 20 h. Upper panels show mature root segments of wild-type, *pdr2* and *lpr1lpr2* seedlings, lower panels depict the root meristem and EZ, which shows early differentiation of root hairs under-Pi. Scale bar, 200 μm. (PDF 45 kb) Additional file 13: Table S11. Protein/transcript list of spCCA analysis. Shown is the list of mRNAs/proteins that are highly relevant (high weight) within the three canonical variables found in the spCCA analysis. Values in tables illustrate the relative weight of each mRNA/protein. Negative values indicate that these mRNAs/proteins are anti-correlated to the pattern of the respective canonical variable as shown in Fig. 3 and Additional file 14: Figure S3. (XLSX 26 kb) Additional file 14: Figure S3. spCCA analysis. (A) Shown are the experimental design factors used for the supervised correlation analysis. (B, C, and D) Canonical variables (CV) of the spCCA analysis representing a subset of transcripts/proteins that showed maximum correlation with the illustrated patterns generated by the spCCA algorithm. CVs in B and C are also shown in Fig. 4 (see also Additional file 13: Table S11). (PDF 961 kb) Additional file 15: Figure S4. Fe distribution in *fer* and *irt1* mutant plants. (A) Semi-thin (1 μm) longitudinal sections of Perls/DAB stained root tips of wild-type seedlings after transfer from + Pi to + Pi or –Pi (20 h). Shown are overview (scale bar 100 μm) and detail (scale bar 25 μm) images of the root tip. Arrows indicate punctate Fe storages. (B) Perls/DAB Fe staining of wild-type and *irt1* seedlings. Upper and middle panels show mature and young differentiated root segments, respectively. Lower panels show the root meristem. Scale bar 100 μm. (PDF 1598 kb) Additional file 16: Figure S5. Aniline blue staining on *frd3* roots and citrate application. (A) 4-days-old wild-type and *frd3-7* seedlings were transferred from + Pi to + Pi or –Pi medium for 2 days. Left: Aniline blue (callose) staining. Right: photographs. Scale bar, 200 μm. (B, and C) 4-days-old wild-type seedlings were transferred from + Pi to + Pi or –Pi medium supplemented with increasing concentrations of citrate. (B) Daily increase in primary root growth was measured over 3 days and illustrated in segmented boxes within the bar graph. (±SE, *n ≥* 15). Standard error was calculated from the average total root growth within 3 days. (C) Photograph of wild-type plants that were transferred for 5 days to –Pi medium, supplemented with different citrate concentrations. Each colored spot indicates the position of the root tip after the indicated time point. Scale bar, 1000 μm. (PDF 1948 kb) Additional file 17: Detailed description of protein extraction and LC-MS analysis. (PDF 76 kb)
