# Supplementary material for: Comparative expression profiling reveals a role of the root apoplast in local phosphate response
Source: BMC Plant Biol. 2016 Apr 28;16:106. doi: 10.1186/s12870-016-0790-8 (PMC4849097; doi:10.1186/s12870-016-0790-8)
Supplement: Additional file 2: Figure S1. — Correlation and GO term analysis. (A) Heat map of a hierarchical cluster analysis of the group of 48 transcripts altered in all three genotypes upon Pi-depletion. Relative expression values are shown. (B) Scatter plots presenting pairwise correlation analysis (log2 fold changes) of the 48 commonly regulated genes upon Pi-depletion. FC, fold change. (see also Additional file 1: Table S1). (C) Correlation analysis of a subset of 241 Pi-responsive genes that were differentially regulated in wild-type and pdr2 but not in lpr1lpr2 roots (p ≤ 0.05, Student’s t-test; 0.66 ≥ FC ≥ 1.5). The upper image shows log2 fold changes of all genes upon Pi-starvation. The lower heat map illustrates the same gene set and expressional changes using a color code. (D) GO term analysis of a subset of 1680 genes that were either Pi-responsive in wild-type, pdr2 and/or lpr1lpr2 roots or that were differentially regulated in Pi-replete pdr2 and/or lpr1lpr2 roots (p ≤ 0.05, Student’s t-test; 0.66 ≥ FC ≥ 1.5). Each segment in a wheel represent one GO term. The top five GO terms are listed and significance values are shown. The complete list of genes and GO terms is shown in Additional file 4: Table S3. (see also Additional file 4: Table S3, Additional file 5: Table S4, Additional file 6: Table S5, Additional file 7: Table S6). (PDF 246 kb) [file 12870_2016_790_MOESM2_ESM.pdf]

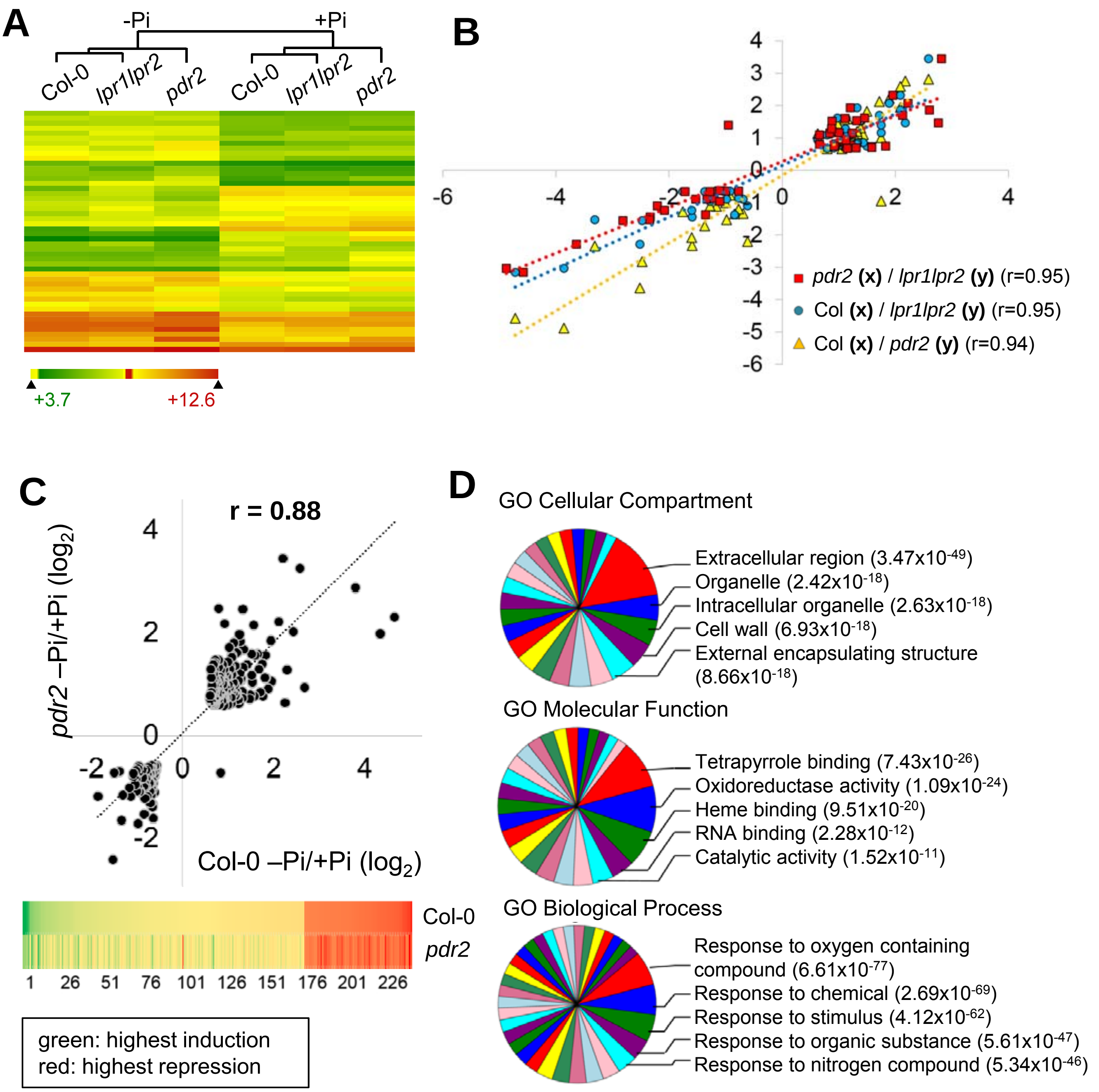

Figure S1. Correlation and GO Term Analysis

(**A**) Heat map of a hierarchical cluster analysis of the group of 48 transcripts altered in all three genotypes upon Pi-depletion. Relative expression values are shown. (**B**) Scatter plots presenting pairwise correlation analysis (log2 fold changes) of the 48 commonly regulated genes upon Pi-depletion. FC, fold change. See also Table S1. (**C**) Correlation analysis of a subset of 241 Pi-responsive genes that were differentially regulated in wild-type and *pdr2* but not in *lpr1lpr2* ($p \leq 0.05$, Student's t-test; $0.66 \geq FC \geq 1.5$). The upper image shows log2 fold changes of all genes upon Pi-starvation. The lower heat map illustrates the same gene set and expressional changes using a color code. (**D**) GO term analysis of a subset of 1,680 genes that were either Pi-responsive in wild-type, *pdr2* and/or *lpr1lpr2* or that were differentially regulated in Pi-replete *pdr2* and/or *lpr1lpr2* roots ($p \leq 0.05$, Student's t-test; $0.66 \geq FC \geq 1.5$). Each segment in a wheel represent one GO term. The top five GO terms are listed and significance values are shown. The complete list of genes and GO terms is shown in Table S3. See also Table S3-S6
