# Supplementary material for: Comparative expression profiling reveals a role of the root apoplast in local phosphate response
Source: BMC Plant Biol. 2016 Apr 28;16:106. doi: 10.1186/s12870-016-0790-8 (PMC4849097; doi:10.1186/s12870-016-0790-8)
Supplement: Additional file 12: Figure S2. — Fe staining and root growth assay. Perls/DAB Fe staining on 4-days-old seedlings that were transferred from + Pi to + Pi or –Pi medium for 20 h. Upper panels show mature root segments of wild-type, pdr2 and lpr1lpr2 seedlings, lower panels depict the root meristem and EZ, which shows early differentiation of root hairs under-Pi. Scale bar, 200 μm. (PDF 45 kb) [file 12870_2016_790_MOESM12_ESM.pdf]

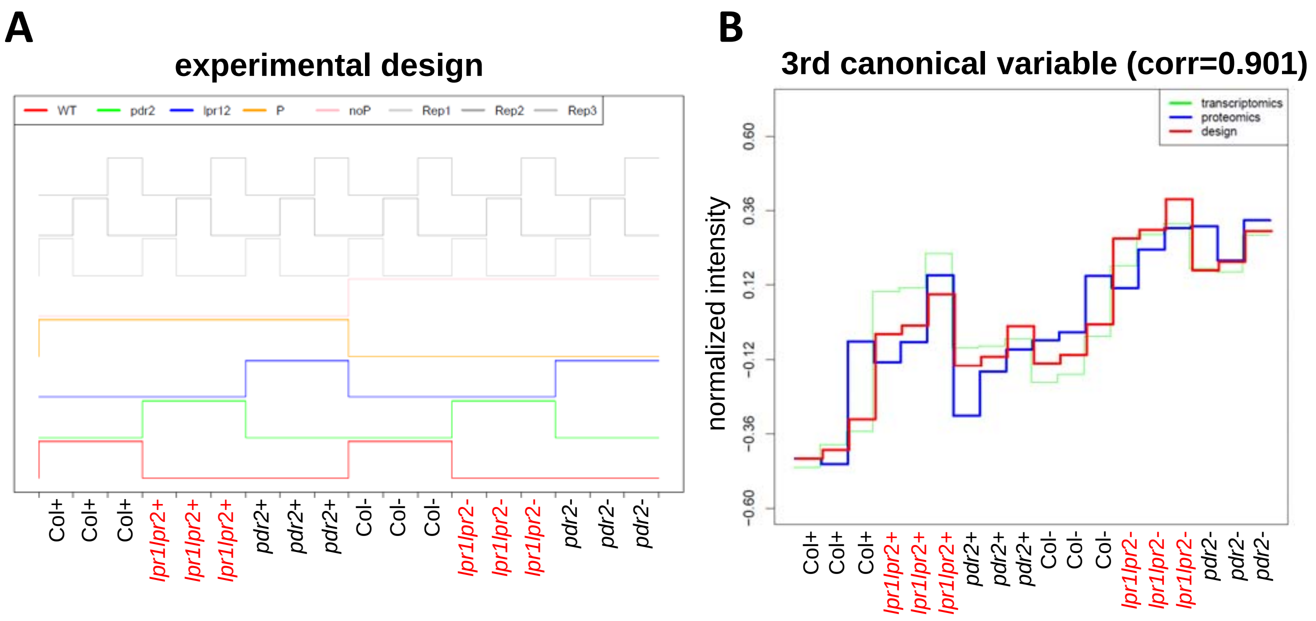

Figure S2. spCCA Analysis.

(A) Shown are the experimental design factors used for the supervised correlation analysis. **(B)** The third Canonical variable (CV) of the spCCA analysis representing a subset of transcripts/proteins that showed maximum correlation with the illustrated patterns generated by the spCCA algorithm.
