# Supplementary material for: Comparative expression profiling reveals a role of the root apoplast in local phosphate response
Source: BMC Plant Biol. 2016 Apr 28;16:106. doi: 10.1186/s12870-016-0790-8 (PMC4849097; doi:10.1186/s12870-016-0790-8)
Supplement: Additional file 14: Figure S3. — spCCA analysis. (A) Shown are the experimental design factors used for the supervised correlation analysis. (B, C, and D) Canonical variables (CV) of the spCCA analysis representing a subset of transcripts/proteins that showed maximum correlation with the illustrated patterns generated by the spCCA algorithm. CVs in B and C are also shown in Fig. 4 (see also Additional file 13: Table S11). (PDF 961 kb) [file 12870_2016_790_MOESM14_ESM.pdf]

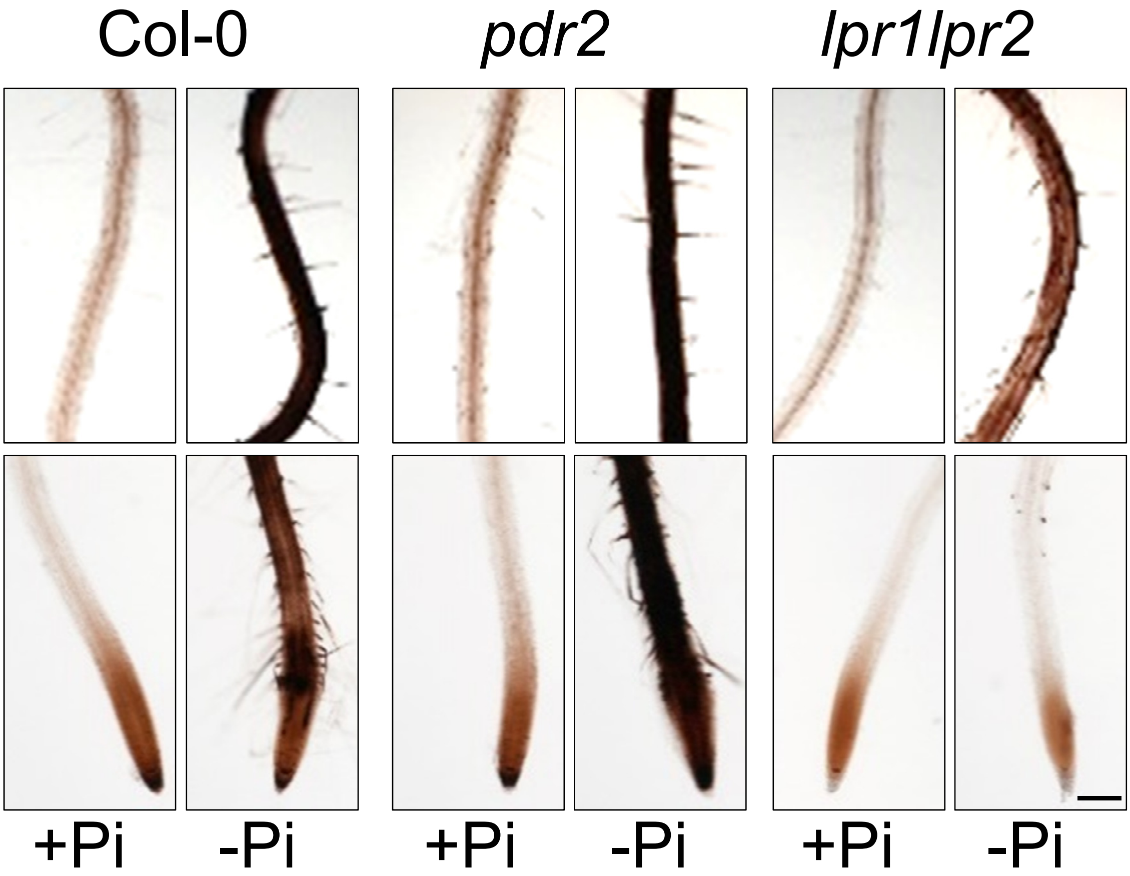

Figure S3. Fe Staining and Root Growth Assay.

Perls/DAB Fe staining on 4-days-old seedlings that were transferred from +Pi medium to +Pi or –Pi medium for 20 hours. Upper panels show mature root segments of wild-type, *pdr2* and *lpr1lpr2* seedlings, lower panels depict the root meristem and EZ, which shows early differentiation of root hairs under-Pi. Scale Bar, 200 µm.
