# Supplementary material for: Comparative expression profiling reveals a role of the root apoplast in local phosphate response
Source: BMC Plant Biol. 2016 Apr 28;16:106. doi: 10.1186/s12870-016-0790-8 (PMC4849097; doi:10.1186/s12870-016-0790-8)
Supplement: Additional file 15: Figure S4. — Fe distribution in fer and irt1 mutant plants. (A) Semi-thin (1 μm) longitudinal sections of Perls/DAB stained root tips of wild-type seedlings after transfer from + Pi to + Pi or –Pi (20 h). Shown are overview (scale bar 100 μm) and detail (scale bar 25 μm) images of the root tip. Arrows indicate punctate Fe storages. (B) Perls/DAB Fe staining of wild-type and irt1 seedlings. Upper and middle panels show mature and young differentiated root segments, respectively. Lower panels show the root meristem. Scale bar 100 μm. (PDF 1598 kb) [file 12870_2016_790_MOESM15_ESM.pdf]

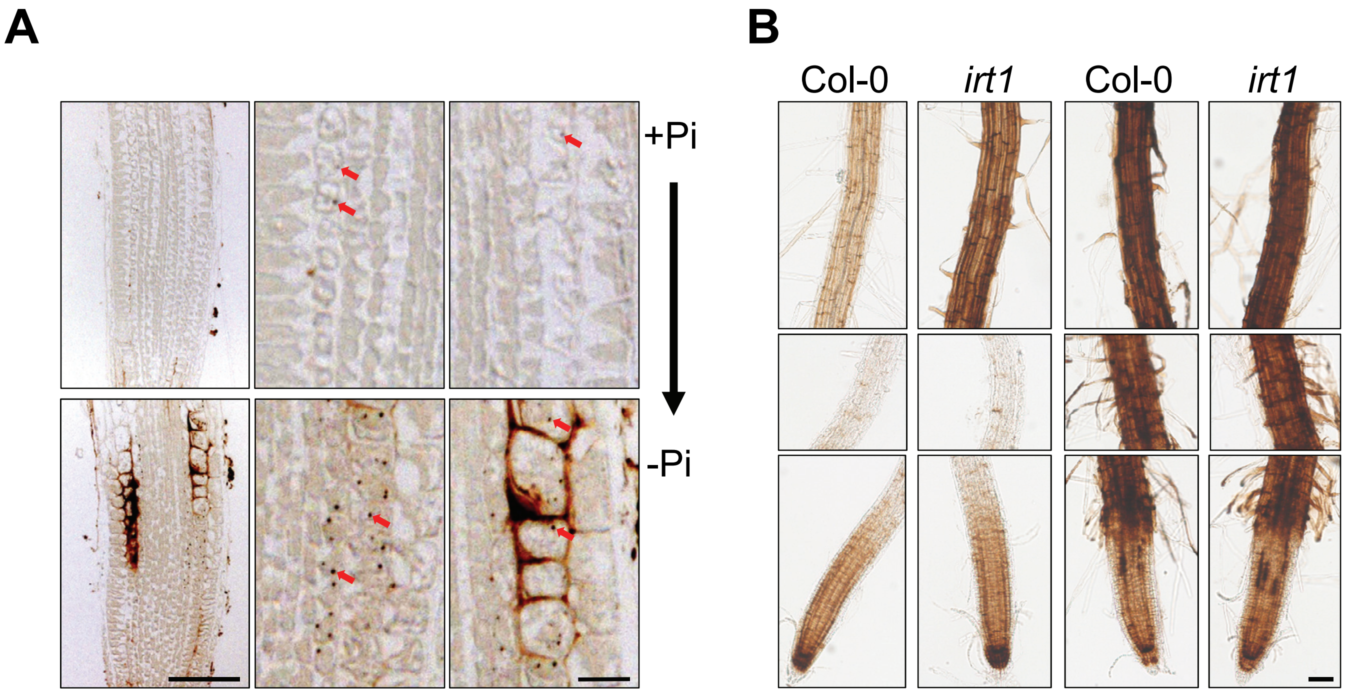

Figure S4. Fe Distribution in *fer* and *irt1* Mutant Plants.
(A) Semi-thin (1 µm) longitudinal sections of Perls/DAB stained root tips of wild-type seedlings after transfer from +Pi to +Pi or –Pi (20h). Shown are overview (scale bar 100 µm) and detail (scale bar 25 µm) images of the root tip. Arrows indicate punctate Fe storages. (B) Perls/DAB Fe staining of wild-type and irt1 seedlings. Upper and middle panels show mature and young differentiated root segments, respectively. Lower panels show the root meristem. Scale bar 100 µm.
