# Supplementary material for: Comparative expression profiling reveals a role of the root apoplast in local phosphate response
Source: BMC Plant Biol. 2016 Apr 28;16:106. doi: 10.1186/s12870-016-0790-8 (PMC4849097; doi:10.1186/s12870-016-0790-8)
Supplement: Additional file 16: Figure S5. — Aniline blue staining on frd3 roots and citrate application. (A) 4-days-old wild-type and frd3-7 seedlings were transferred from + Pi to + Pi or –Pi medium for 2 days. Left: Aniline blue (callose) staining. Right: photographs. Scale bar, 200 μm. (B, and C) 4-days-old wild-type seedlings were transferred from + Pi to + Pi or –Pi medium supplemented with increasing concentrations of citrate. (B) Daily increase in primary root growth was measured over 3 days and illustrated in segmented boxes within the bar graph. (±SE, n ≥ 15). Standard error was calculated from the average total root growth within 3 days. (C) Photograph of wild-type plants that were transferred for 5 days to –Pi medium, supplemented with different citrate concentrations. Each colored spot indicates the position of the root tip after the indicated time point. Scale bar, 1000 μm. (PDF 1948 kb) [file 12870_2016_790_MOESM16_ESM.pdf]

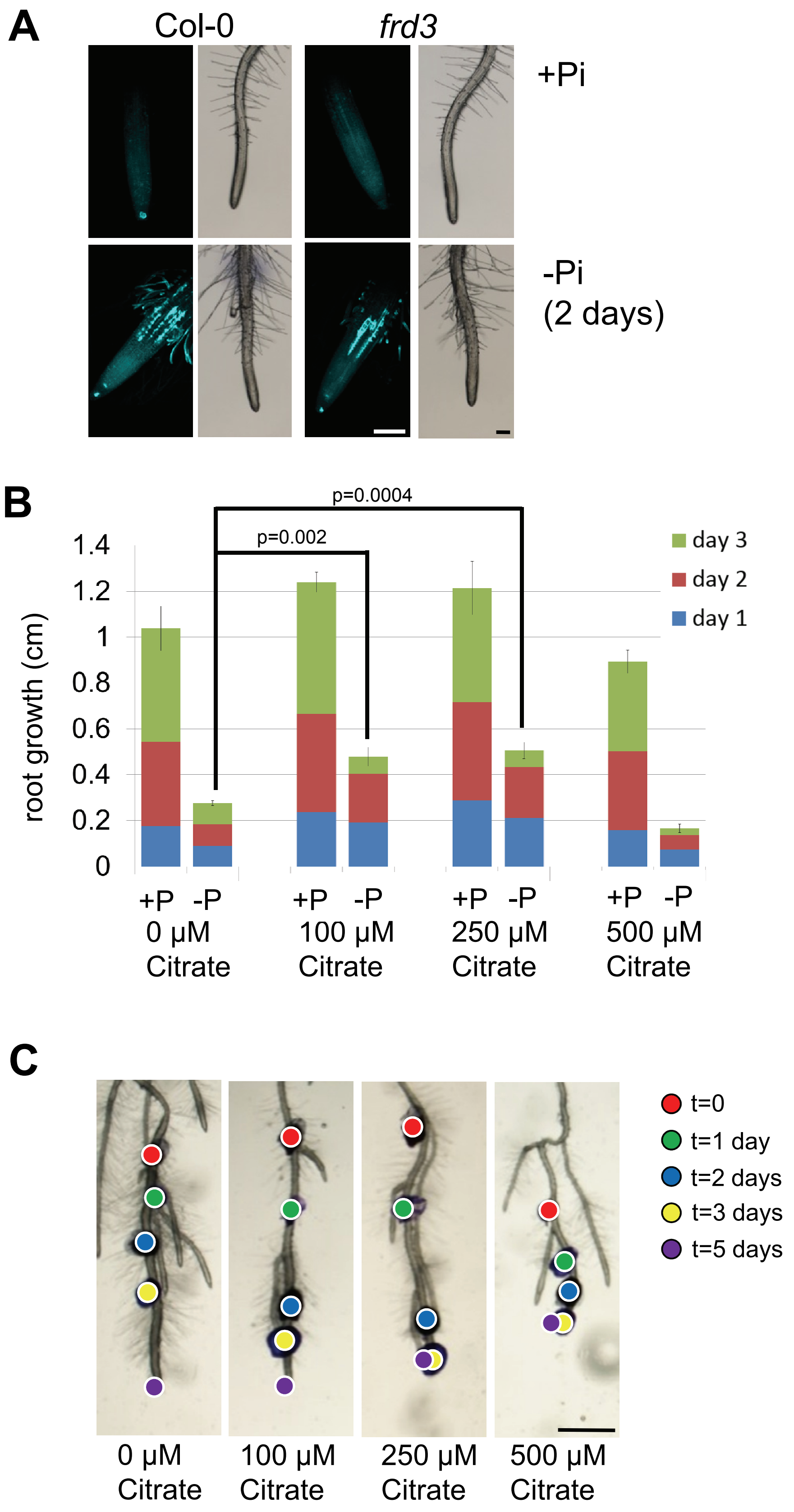
A
Col-0
frd3
+Pi
-Pi
(2 days)
B
p=0.0004
p=0.002
root growth (cm)
1.4
1.2
1
0.8
0.6
0.4
0.2
0
day 3
day 2
day 1
+P -P
0 μM Citrate
+P -P
100 μM Citrate
+P -P
250 μM Citrate
+P -P
500 μM Citrate
C
t=0
t=1 day
t=2 days
t=3 days
t=5 days
0 μM Citrate
100 μM Citrate
250 μM Citrate
500 μM Citrate

Figure S5. Aniline Blue Staining on *frd3* Roots and Citrate Application.

(A) 4-days-old wild-type and *frd3-7* seedlings were transferred from +Pi medium to +Pi or –Pi medium for 2 days. Left: Aniline blue callose staining. Right: photographs. Scale bar, 200 µm. **(B-C)** 4-days-old wild-type seedlings were transferred from +Pi medium to +Pi or –Pi medium supplemented with increasing concentrations of citrate. **(B)** Daily increase in primary root growth was measured over 3 days and illustrated in segmented boxes within the bar graph. (±SE, n≥15). Standard error was calculated from the average total root growth within 3 days. **(C)** Photograph of wild-type plants that were transferred for 5 days to –Pi medium, supplemented with different citrate concentrations. Each colored spot indicates the position of the root tip after the indicated time point. Scale bar, 1000µm.
