# Supplementary material for: Comparative expression profiling reveals a role of the root apoplast in local phosphate response
Source: BMC Plant Biol. 2016 Apr 28;16:106. doi: 10.1186/s12870-016-0790-8 (PMC4849097; doi:10.1186/s12870-016-0790-8)
Supplement: Additional file 17: — Detailed description of protein extraction and LC-MS analysis. (PDF 76 kb) [file 12870_2016_790_MOESM17_ESM.pdf]

Additional File 17: Methods

Protein Extraction

Proteins were extracted from 100 mg of root tissue and ground to a fine powder under liquid nitrogen. The tissue was suspended in 300 µl of ice-cold extraction buffer (100 mM Tris-HCL, pH 8.5; 1% (w/v) SDS; 5% glycerol (v/v); 5 mM EDTA; 0.5% ß-mercaptoethanol added fresh (v/v); 1% Protease Inhibitor Cocktail for Plant Cell Lysate (SIGMA Product No. P9599, v/v, added fresh)) and mixed vigorously for 30 min at 4°C. 300 µl of water-saturated phenol were added and mixing was prolonged for 30 minutes. The suspension was centrifuged at 10,000 g at 4°C for 10 min. The phenol phase was transferred to a new reaction tube and an equal volume of re-extraction buffer (100 mM Tris-HCl, pH 8.5; 20 mM KCl; 10 mM EDTA; 0.5% ß-mercaptoethanol added fresh(v/v)) was added. The homogenate was mixed vigorously for 15 min at 4°C and centrifuged as above. Proteins were precipitated from the phenol phase at -20°C overnight by adding 10 volumes of 100 mM ammonium acetate in methanol. Precipitated proteins were collected by centrifugation at 6,000 g at 4°C for 10 min. The protein pellet was washed twice with 10 ml 20% 50mM ammonium bicarbonate, 80% acetone and air dried for 30 minutes at room temperature. The protein pellet was dissolved in 100 µl of 8 M urea, 50 mM ammonium bicarbonate and the protein content was determined with the 2D-Quant kit (GE Healthcare) according to the instructions. Disulfide bonds were reduced with 200 mM dithiothreitol (DTT), 100 mM Tris-HCl and alkylated with an excess of 200 mM iodoacetamide (IAA), 100 mM Tris-HCl. Proteins were digested with trypsin at an enzyme to protein ratio of 1:50 at 37°C overnight. The protein digest was desalted using reverse phase solid phase extraction chromatography in STAGE tips. The C18 matrix was conditioned with 80% acetonitrile, 0.1% FA in ddH2O and equilibrated with 0.1% FA in

ddH2O. Bound peptides were washed with 0.1% FA in ddH2O, eluted with 80% acetonitrile, 0.1% FA in ddH2O and dried to completion in a vacuum concentrator. The peptides were dissolved in 5% acetonitrile, 0.1% TFA.

Liquid Chromatography and Mass Spectrometry

The samples were measured in random order. One µg of peptides were injected into an EASY-nLC II nano liquid chromatography system (Thermo Fisher Scientific). Peptides were separated using C18 reverse phase chemistry employing a pre-column (EASY column SC001, length 2 cm, ID 100 µm, particle size 5 µm) in line with an EASY column SC200 with a length of 10 cm, an inner diameter (ID) of 75 µm and a particle size of 3 µm (both from
